# Body mass index and dental caries in young people: a systematic review

**DOI:** 10.1186/s12887-019-1511-x

**Published:** 2019-04-23

**Authors:** Martha Paisi, Elizabeth Kay, Cathy Bennett, Irene Kaimi, Robert Witton, Robert Nelder, Debra Lapthorne

**Affiliations:** 1 Faculty of Medicine and Dentistry, University of Plymouth, Peninsula Dental School, room C507, Portland Square, Plymouth, Plymouth, Devon PL4 8AA UK; 2 Office of Research and Innovation, Royal College of Surgeons in Ireland, Dublin, Ireland; 3 School of Computing, Electronics and Mathematics, Plymouth University, Plymouth, PL4 8AA UK; 4 Office of the Director of Public Health, Plymouth City Council, Plymouth, PL6 5UF UK; 5 Public Health England, South West, Follaton House, Plymouth Road, Totnes, Devon TQ9 5NE UK

**Keywords:** Obesity, Caries, Children, Adolescents

## Abstract

**Background:**

Obesity and caries in young people are issues of public health concern. Even though research into the relationship between the two conditions has been conducted for many years, to date the results remain equivocal. The aim of this paper was to determine the nature of the relationship between Body Mass Index (BMI) and caries in children and adolescents, by conducting a systematic review of the published literature.

**Methods:**

A systematic search of studies examining the association between BMI and caries in individuals younger than 18 years old was conducted. The electronic bibliographic databases PubMed, MEDLINE, Embase, CINAHL, CENTRAL and Google Scholar were searched. References of included studies were checked to identify further potential studies. Internal and external validity as well as reporting quality were assessed using the validated Methodological Evaluation of Observational Research checklist. Results were stratified based on the risk of flaws in 14 domains 10 of which were considered major and four minor.

**Results:**

Of the 4208 initially identified studies, 84 papers met the inclusion criteria and were included in the review; conclusions were mainly drawn from 7 studies at lower risk of flaws. Three main types of association between BMI and caries were found: 26 studies showed a positive relationship, 19 showed a negative association, and 43 found no association between the variables of interest. Some studies showed more than one pattern of association. Assessment of confounders was the domain most commonly found to be flawed, followed by sampling and research specific bias. Among the seven studies which were found to be at lower risk of being flawed, five found no association between BMI and caries and two showed a positive association between these two variables.

**Conclusions:**

Evidence of an association between BMI and caries was inconsistent. Based on the studies with a low risk lower risk of being flawed, a positive association between the variables of interest was found mainly in older children. In younger children, the evidence was equivocal. Longitudinal studies examining the association between different indicators of obesity and caries over the life course will help shed light in their complex relationship.

**Electronic supplementary material:**

The online version of this article (10.1186/s12887-019-1511-x) contains supplementary material, which is available to authorized users.

## Background

Obesity and caries are important issues of public health concern and affect a large number of children and adolescents worldwide [1, 2]. Both can have adverse impacts on wellbeing and quality of life and are associated with significant costs to the society [1, 3]. A number of research studies have investigated the relationship between weight status and caries, largely because health problems associated with growth and development and with oral disease may share a common pathway through dietary behaviours [4, 5]. Whilst some studies have indicated that there is a link between body weight in children and caries development, the results are mixed and conflicting.

A few studies have shown that increased weight status is associated with a higher burden of dental caries [6]. Others have shown that lower weight status is associated with greater caries experience [7, 8]. There are also several reports which did not find evidence of an association between the two variables [5, 9]. Therefore, the direction and effect size of the relationship between obesity and caries have not yet been established and there is a need to systematically review reports of studies that provide data on these two conditions.

When this research commenced, four systematic reviews examining the association between weight status and caries had been conducted [10–13]. The first review which included studies examining the relationship of obesity and caries in children, adolescents and adults found that only three studies provided high quality evidence [11]. The results of these studies were conflicting and as such the authors of the review suggested that no clear conclusions could be drawn. In another systematic review, Hayden et al. [13] reported that a significant association was evident between obesity and caries in individuals less than 18 years of age and that this relationship was moderated by age and socioeconomic status. When the meta-analysis included studies that used standardised assessments of obesity, a positive, albeit weak relationship was identified between the variables of interest, but only in the permanent teeth. The review by Hooley et al. [10] which included studies conducted with children and adolescents, found that both high and low BMI related to higher burden of caries, but pointed out that the results of the primary studies were not consistent. The latest systematic review [12] was not able to draw any conclusions from the evidence available nor could it establish the impact of any confounders or effect modifiers on the association between obesity and caries in children and adolescents.

As well as having mixed results, these systematic reviews used their own, non-validated or non-study design specific tools to assess the methodological quality of published papers and they appraised evidence that was collected at different times. Therefore, taking into consideration the methodological gaps in the literature and the fact that the relationship between weight status and caries remains inconclusive, a systematic review using a standardised quality assessment tool was required.

### Objectives

The purpose of this systematic review was to examine and update the evidence about an association between BMI and dental caries in children and adolescents, using a validated and study-design specific tool. The review also aimed to identify gaps in the literature in order to offer recommendations for future research.

## Methods

The research protocol was set a priori and can be accessed by contacting the corresponding author. The PECOs framework (i.e. Population, Exposure, Comparison, Outcome, Study design) was used to structure the search strategy and further details are provided below.

The literature searches were undertaken in July 2014 and were limited to articles published after 1980. The year 1980 was chosen as a starting point in the review, as there has been a significant rise in childhood obesity since that year [14]. The search strategy included synonyms related to the main outcomes (caries and weight status) as well as the population of interest (children and adolescents). An example of the search strategy approach is presented below (Table 1).

**Table 1:** PubMed search strategy

Search	Query
#1	(Overweight OR obes* OR underweight OR BMI OR “body mass” OR adiposity OR weight OR “body size” OR waist OR hip OR skinfold* OR Maln*)
#2	(caries OR “dental health” OR “primary dentition” OR “oral health” OR decay OR cavities OR dmf* OR dft OR dfs)
#3	(child* OR preschool OR pediatr* or paediatr* OR minor OR pupil* OR Toddler* OR adolesc* OR teen* OR “young person” OR “young people” OR youth)
#4	#1 AND #2 AND #3 Filters: Publication date from 1980/01/01 to 2014/07/16; English

The databases searched were PubMed, EBSCO MEDLINE, Ovid Embase, EBSCO CINAHL and CENTRAL through the Cochrane Library. Google Scholar was also searched and the references of included studies were manually checked for additional studies. Grey literature (such as PhD theses, technical/governmental reports and conference proceedings), studies published in languages other than English and those whose full text was not accessible were excluded from the review due to budget restrictions.

The inclusion and exclusion criteria which were set a priori are listed and explained in Table 2.

**Table 2:** Inclusion and exclusion criteria

Inclusion criteria	
Dental caries measured by differences in the number of teeth or surfaces that were decayed, missing, filled or presence/absence of caries BMI objectively measured The relationship between caries and BMI examined in individuals less than 18 years old Observational studies analysing primary or secondary data	
Exclusion criteria	
Adult population (> 18 years old) No exclusions on gender or ethnicity Did not assess dental caries, BMI or the association between the two Self-reported measures of BMI Narrative reviews, case reports, letters and editorials Animal studies Grey literature Studies published in languages other than English	

All identified titles/abstracts were then imported electronically into the bibliographic database Endnote (version X7.2). Following deduplication, the titles/abstracts of the identified papers were screened for inclusion and then the full text of selected papers was reviewed by two independent reviewers (MP, EK) for inclusion or exclusion. Where the reviewers did not agree, the paper was jointly reviewed against the specific criteria and consensus was reached. A data extraction form which had previously been pilot-tested by the research team (MP and EK) on four relevant papers was used to extract details of individual studies. Thereafter the basic data were summarised in a table format [(i.e. city and country, setting, study design, sample size and gender distribution, age group, HDI category, BMI classification and caries measure, type of relationships identified between BMI and caries (main summary measures included odds ratio, risk ratio, difference in means)]. The data extraction was conducted by two independent researchers (MP, EK) and in case of disagreement, a discussion was held to reach consensus. Due to the extensive time period covered and for the purpose of consistency, no contact with the investigators was sought.

Owing to the nature of research question, the studies examined were of observational design. Critical appraisal of the studies included in the review was conducted by two independent reviewers (MP, CB) and was based on the validated tool “Methodological Evaluation of Observational Research Checklist” (MEVORECH) [15]. The criteria and research specific flaws for the assessment of study quality against specific domains were set a priori by the research team. For the purpose of this review, BMI was considered as the exposure and dental caries as the outcome. Diet and socioeconomic status were considered as the main covariates which could affect the relationship between BMI and caries. The risk of flaw in each study was evaluated against ten major and four minor domains of internal and external validity and reporting [16]. The risk of flaws in each domain was categorised as low, high or unclear.

The major domains were:

*Definition of exposure* - whether BMI classification status was assessed: high risk if intensity/dose was not assessed or not reported;

*Source of exposure data* - whether the information was obtained from medical or administrative records for healthcare purposes, or obtained from registries where data were collected for epidemiologic evaluation or assessed by researchers specifically for the study: the domain was considered to be at high risk when the information was obtained from medical or administrative records and no information on data collection methods and analysis was provided;

*Assessment of outcome* - the source used to measure the outcome and the validity of the outcome measure: the domain was at high risk of being flawed when the information was obtained from medical or administrative records or when unvalidated tools were used to measure the outcome;

*Reliability of exposure estimates* - whether intra/inter observer variability was assessed objectively and acceptable values were achieved: the domain was at high risk of being flawed when variability was assessed subjectively or was lower than pre-determined levels (kappa value for inter observer and/or intra observer reliability < 0.80 and/or < 0.90, respectively);

*Reliability of outcome estimates* -. same criteria as reliability of exposure estimates; *Confounder assessment* -. whether the major confounders were assessed and whether valid tools were used to measure them-the domain was at high risk of being flawed if one factor had a high risk of flaw or if both factors had an unclear risk of flaw;

*Sampling bias* - a. the sampling of the population-this factor was at high risk of flaw when the study used a convenience sample with or without randomisation; b. whether sampling bias was addressed in the analysis via weighting, post-stratification or other methods, and c. the response rate, with an acceptable response rate set at above 80%. High risk in this domain was assigned if one of the above factors was at high risk of flaw or two factors had an unclear risk of being flawed;

*Research specific bias* - the methods used to reduce research specific bias e.g. standardisation, whether dose-response was assessed in the analysis and whether sample size included a power calculation. The domain was at high risk of flaw if one of the factors above had a high risk, or two factors had an unclear risk of flaw;

*Exclusion bias* -. the total exclusion rate from the analysis-the domain was at high risk of being flawed when the exclusion rate from the analysis was greater than 25%;

*Attrition bias* (applicable to longitudinal and case-control studies)- the total loss to follow up drop out difference of dropout among the groups. The domain was at high risk of flaw when total loss to follow up was greater than or equal to 20% or when drop out among the groups differed by more than 10% or when the reasons for participants withdrawal were not the same for the two groups.

The minor domains included:

*Funding* -. the source of funding and the role of sponsors in data analysis and interpretation. The domain was at high risk of being flawed if the study was funded by the industry or through a combined industry-grant source and it was not clear whether the sponsors were involved in data analysis and interpretation or when the sponsors were involved in data analysis and interpretation;

*Conflict of interest*: the domain was at high risk of flaw if a conflict of interest was reported for any of the authors and if the two reviewers considered the declared interest to be conflicting;

*Blinding* - masking of exposure for the researchers who assessed the outcome. The domain was at high risk of flaw if the assessors were aware of the child’s BMI status;

*Selective reporting of results*: high risk of flaw when there was incomplete or selective reporting of the tested hypothesis and/or crude estimates only were provided.

Risk of flaws was assessed both at outcome and study level. Although the assessment of study quality was based primarily on the risk of flaw in the main domains, the effect of flaws in minor domains, and how they could affect the overall quality of the study were also taken into consideration. The conclusions of this review are primarily based on the findings of studies found to be at lower risk of being flawed.

The Preferred Reporting Items for Systematic Reviews and Meta-Analyses (PRISMA) statement was used to report the present review [17].

## Results

**Fig. 1:**
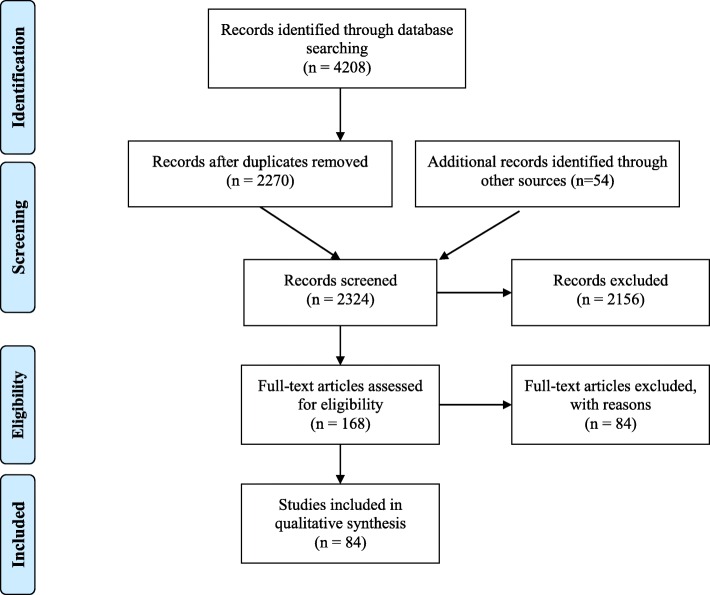
PRISMA Flow Diagram of search results. *Modified from:* Moher D, Liberati A, Tetzlaff J, Altman DG, The PRISMA Group (2009). *P*referred *R*eporting *I*tems for *S*ystematic Reviews and *M*eta-*A*nalyses: The PRISMA Statement. PLoS Med 6 (7): e1000097

Figure 1 is the PRISMA Flow Diagram of search results [17].

The initial search retrieved 4208 potential studies. After the duplicates were removed, 2270 studies remained. Another 54 papers that were identified from other sources were added. Of these studies, 2156 were excluded on title or abstract as they did not meet inclusion criteria. Of the remaining papers (*N* = 168), 84 were excluded after reading the full text. Reasons for exclusion were recorded. A list of the excluded articles together with the reasons of exclusion is provided in Additional file 1. Eighty four papers met the inclusion criteria and were included in the systematic review.

### Descriptive characteristics

The characteristics of all studies incorporated in the review are summarised in Additional file 2. The countries where the studies took place were categorised into four levels of development based on the Human Development Index (HDI) which merges life expectancy, educational attainment and income into a single score and which differentiates levels of ‘human development’ across different countries (i.e. very high, high, medium and low development) [(Human Development Report Statistical Tables 2014 [18]]. Thirty nine studies were conducted in very high human development (HD) countries, 28 in high-, 14 in medium-and three in low HD countries.

The majority of the 84 included studies were of cross-sectional design (*N* = 74). Eight studies were of case control design and two of longitudinal design. The age of the participants was between one and 18 years. The number of participants in the studies ranged from 55 to 10,180. The studies that had the highest sample sizes were those that used secondary data in their analysis from nationally representative surveys in the United States (i.e. National Health and Examination Survey (NHANES). Eighteen studies had a sample population of less than 200 people. The majority of the studies were conducted in schools, whilst a small number took place in dental clinics/department, mobile offices/households and child welfare centers.

Various classification systems were used in the assessment of obesity. The majority of studies used the BMI-for-age centiles from the 2000 Centers for Disease Control and Prevention [19] and the BMI for age z-scores [20]. Others employed the international age and gender data sets recommended by the International Obesity Task Force [21] and few used the BMI z-scores. There were also some that used national growth references.

Dental caries was evaluated in the studies mainly through visual examination of teeth or tooth surfaces using the WHO criteria [22], although eight studies (please see Additional file 2) used radiographic examination in addition to the visual examination. In some studies, radiographs were taken into consideration only under certain conditions.

Three main types of association between BMI and caries were found: 26 studies showed a positive relationship, 19 showed a negative association, and 43 found no association between the variables of interest. Some studies showed more than one pattern of association.

### Critical appraisal

Table 3 presents the level of risk of flaw across studies per outcome measure.

**Table 3:** Findings on risk of flaws per outcome (as derived from MEVORECH)

Domains	High risk Number of studies, (%)	Low risk Number of studies, (%)	Unclear risk Number of studies, (%)	Not applicable Number of studies, (%)
Exposure definition	4 (4.8)	80 (95.2)	0	0
Assessment of exposure	10 (11.9)	73 (86.9)	1(1.2)	0
Assessment of outcome	17(20.2)	66(78.6)	1(1.2)	0
Reliability of exposure estimates	1(1.2)	5(6.0)	78(92.9)	0
Reliability of outcome estimates	5(6.0)	37(44)	42(50)	0
Confounder assessment	71(84.5)	2(2.4)	11(13.1)	0
Sampling bias	57(67.9)	25(29.8)	2(2.4)	0
Research specific bias	42(50.0)	38(45.2)	4(4.8)	0
Exclusion bias	2(2.4)	26(31)	56(66.7)	0
Attrition bias	2(2.4)	4(4.8)	3(3.6)	75(89.3)
Funding	4(4.8)	10(11.9)	70(83.3)	0
Conflict of interest	0	30(35.7)	54(64.3)	0
Blinding	0	8(9.5)	76(90.5)	0
Selective reporting of results	33(39.3)	48(57.1)	3(3.6)	0

None of the studies included in the review were found to have a low risk of flaw in all major and minor domains.

Seventy seven of the 84 studies were found to have a high risk of flaw in one or more of major domains and 37 were to have a high risk of flaw in at least one minor domain. With regard to the main domains, high risk of flaw was most common in the domains of confounder assessment (71/84), sampling bias (56/84) and research-specific bias (43/84). Interestingly, only two studies were found to have a low risk of flaw in the confounder domain, while for 11 studies this was unclear. The majority of the studies that assessed the main confounders failed to report whether they used validated tools to assess them. In the minor domains, high risk of flaw was most common in the selective reporting of results (39%).

The risk of flaw in each domain across studies and for each individual study can be found in Additional file 3. Only seven studies were judged as not having a high risk of being flawed in any of the key domains [5, 23–28]; however, the risk of flaw in some of the domains was unclear. Of these seven low risk studies, five found no association between dental caries and BMI [5, 23–26]. Two studies found a positive association between the two variables of interest and both were conducted in India [27, 28].

Honne et al. [27] found a significant positive association between BMI, decayed teeth (DT) and the sum of decayed, missing and filled teeth (DMFT) in 463 adolescents aged 13 to 15 years. The study also showed that the risk of caries in overweight/obese individuals was 3.68 times higher in overweight/obese individuals compared to those who were low/normal weight. Sakeenabi et al. [28] examined a cohort of 1550 school age children and found that in 6 year old children who were overweight or obese, the risk of caries was 1.92 and 3.6 times higher compared to those of normal weight. The risk of caries in 13 year olds who were overweight and obese was 1.68 and 1.8 times higher, respectively, than in the normal weight children.

The remaining studies included in the systematic review (those which were found to have one or more key domains at high risk of being flawed) (*N* = 77), most commonly found no association between BMI and dental caries (*N* = 38). However, some found a positive association (*N* = 24) between dental caries and BMI and others found a negative association (*N* = 19). The latter association was not evident in the studies which were found to be at lower risk of being flawed. The age ranges of children in each category can be seen in Additional file 3.

The significant statistical, clinical and methodological hetereogeneity among the studies that were evaluated, precluded a quantitative analysis of the findings. Sources of hetereogeneity could be: (i) different effect measures used (e.g. odds ratios, mean difference, prevalence ratios etc); (ii) Sample characteristics (e.g. age, country etc); (iii) differences in sampling methodology with some studies involving some form of random sampling and others simply convenience sampling complicated by the fact that no consistency of the effect measures existed among the groups of studies. Furthermore, some studies used secondary data analysis from large national health data sets (i.e. NHANES) and these are fundamentally different from the other primary studies which set out to try to observe the effect of BMI on caries; (iv) different settings: Although the majority of studies took place in the school setting and involved healthy participants, there were others that were conducted in dental clinics/departments; (v) data collection tools and diagnostic or classification criteria; (vi) different statistical analyses employed. e.g., a point that also was raised by a previous systematic review [10], is that the relationship was not commonly examined on the whole spectrum of BMI and sometimes it was unclear whether children who were underweight were excluded from the analyses or were merged into the normal-weight category; (vii) different levels of risk of flaws among the studies.

## Discussion

The current systematic review provides updated information on the association between weight status (as determined by BMI) and caries in children and adolescents using a validated and study design specific tool. Although it was not possible to pool the results in a quantitative manner (meta-analysis) due to the presence of significant heterogeneity as discussed earlier, this review has highlighted the complexity of the relationship between the two variables and identifies key methodological problems regarding the issue.

As in other systematic reviews [10–13, 29], the current review indicated that the evidence of an association between BMI and caries was mixed and not consistent. Two out of the seven less flawed studies included in the review found a positive association between BMI and caries. Both were conducted in India. Hooley et al. [10] have previously reported that studies which identified a positive relationship between BMI and caries took place mostly in the US and Europe. This may be explained by the increase in affluence observed in economies such as India in recent years, which is accompanied by increased obesity rates as well as energy and fat intake [30]. Increasing levels of physical inactivity may also have a role in the observed patterns [31]. In addition, caries levels in developing countries are increasing due to increased sugar consumption [32]. Thus, the rapidly changing world economy and consequent changes in lifestyle seem to be affecting both the prevalence of obesity and caries, and the pattern of association between them, but this change appears to be evident only in certain developing countries such as India.

Five studies with the lowest levels of flaws included in the review showed no relationship between BMI and caries [5, 23–26]. Given the known association of diet (i.e. sugar consumption) with both conditions, this observed lack of association suggests that diet may affect the two conditions in different ways. The studies with the lowest risk of flaws which found a positive association between BMI and caries [27, 28] assessed the relationship mainly in the permanent dentition. The literature indicates that age influences the relationship between obesity and caries and an association is more easily observed in older children than in the very young i.e. the association between BMI and caries is stronger and more consistent for the permanent dentition [13]. This is probably because both conditions are slowly cumulative across the life course. Future longitudinal studies should therefore examine the relationship in different age groups as well as explore possible mechanisms by which age may account for difference in findings.

Several plausible mechanisms have been proposed for the increasing prevalence and or severity of caries in overweight/obese individuals. The main one relates to diet and particularly high consumption of fermentable carbohydrates (i.e. sugar). Taking into account that the diets of overweight individuals are characterised by a high consumption of fermentable carbohydrates [10] and that sugar is widely recognised as an aetiological factor in caries development [33], this mechanism seems highly possible. Another biological mechanism that could link obesity and caries is the reduced stimulated saliva flow that has been found among obese teenagers when compared to their healthy peers [34]. Reduced saliva flow affects the development of caries and thus obese children could be at higher risk of caries due to low saliva flow. The present review did not seek to explore the mechanisms behind the identified association, however these hypotheses warrant further investigation.

A negative association between BMI and caries (lower BMI, more caries and higher BMI, less caries) was also found, but this was only evident among studies with one or more key domains at high risk of being flawed. One theory about the relationship between caries and underweight suggests that severe untreated dental caries affects eating ability [35]. This hypothesis is supported by the study of Duijister et al. [36] which showed that treatment of severely carious teeth in 48 to 68 months old underweight Philippine children was associated with significant weight gain. As both caries and obesity are multifactorial conditions, the other observed association between low caries and high BMI may be due to an increased consumption of high-fat diets which are positively associated with obesity [37] rather than caries. These findings are further evidence that the relationship between caries and BMI is complex.

This review has identified several factors that appear to be important when examining the relationship between weight status and caries, and these factors may also account for the heterogeneity of results between primary studies. The first important factor is the method of assessing and diagnosing dental caries. Most studies used visual examination of decay, which meant they estimated the level of caries in the population differently from those that used radiographs which have a different diagnostic accuracy. Differences in the methods used to assess caries may therefore have distorted the effect size of a relationship between BMI and caries in some studies [38]. Similarly, there were differences in the BMI classification systems (cut-offs) used in the primary studies and this could have introduced variation in the effect sizes. Previously, it has been shown that the BMI cut-points used have a major impact on the magnitude of effect size in the association between obesity and periodontitis [39]. That is, use of different cut points to identify obesity can introduce considerable heterogeneity between studies. This effect is likely to be similar in obesity/caries studies. These observations highlight the need to use standardised cut-off points to classify obesity and standardised examinations criteria for caries. Doing so would enable comparison of results across studies and the opportunity to statistically meta-analyse worldwide data.

Another factor which can affect the relationship between weight status and caries is the method used to assess weight status. BMI cannot differentiate between fat, muscle or bone mass [5]. However, the evidence of a relationship between obesity and caries is also not consistent when different measures of obesity (e.g. waist circumference, skinfold thickness) or more accurate laboratory methods of body composition assessment (e.g. Dual-energy X-ray Absorptiometry-DXA, air displacement plethysmography) are used [4, 5, 40, 41]. Further studies using different indicators of obesity in different age groups, as well as more accurate methods of assessment may well provide more accurate insights into the real nature of the relationship between obesity and caries. However, whether such studies can be justified is debatable, as their conduct would be extremely expensive.

Confounders are likely to have an important effect on the observed associations and can alter the magnitude of an association and even apparently reverse the direction of the relationship [42]. It was notable that in many of the studies in our review there was an absence of adjustment for confounders and effect modifiers. Even when confounders were assessed, this was only partly done. In addition, different factors were considered as confounders in different studies. This would likely have a profound effect on the findings of several of the primary studies and could therefore affect the type of relationship identified in the evidence synthesis [43]. Research specific and sampling bias were also commonly at high risk of flaw in many of the studies. As these domains can significantly affect the results as well as generalisability of a study, future studies should ensure that appropriate power calculations are conducted prior to the implementation of the study. In addition, appropriate sampling techniques should be used to ensure that the samples are truly representative of the population which the study purports to investigate. Lastly, statistical analyses should always take into account sampling biases and differences in population characteristics.

## Limitations

A meta-analysis was not undertaken due to the significant hetereogeneity between the studies; therefore, it was not possible to quantify the relationship between BMI and caries. The possibility of drawing incorrect conclusions by pooling the results of heterogeneous studies would have been extremely high. Another limitation was that only published and English studies were included and as a result the review is prone to publication and selection bias.

None of the primary studies included in the review were found to have a low risk of flaw at all key domains. However, the validity of our results is enhanced by the decision to draw conclusions only from those studies that were at lower risk of being flawed. As all studies potentially had some flaws, the results should therefore be interpreted with caution.

## Conclusions

Two of the less flawed studies included in the review indicated that BMI and caries were positively related whilst the majority did not find evidence of an association between the two variables. The studies that found positive association were mainly conducted in older children. The present systematic review indicated no evidence of a consistent association between BMI and caries and this finding is in keeping with those of previous systematic reviews.

Well-designed and appropriately powered longitudinal studies examining the relation between different measures of obesity and caries at different life stages are needed. The impact of confounders and effect modifiers should also be thoroughly examined in future studies. Use of standardised diagnostic methods for dental caries and classification of weight status will enable better comparison of the results in the field and thus allow more accurate conclusions to be drawn about the relationship. Sufficient reporting information that would enable other users to adequately draw conclusions on the quality of the primary studies is also warranted.

## Additional files

Additional file 1: Reasons for exclusion of full text articles from the review. This file lists all the full text articles that were excluded from the present review along with the reason for their exclusion. (DOCX 36 kb)

Additional file 2: Characteristics of studies included in the systematic review. This table presents details of all the studies that were included in the review and their citations (end of the table). The studies are grouped according to the type of relationship they identified, with some studies finding more than one pattern of relationship. (DOCX 150 kb)

Additional file 3: Risk of flaws in each individual study and across studies. The table contains the risk of flaws between and within studies against all major and minor domains that were evaluated. The meanings of abbreviations are as follow: L = Low risk of flaw; H = High risk flaw; U = Unclear risk flaw; NA = not applicable. (DOCX 71 kb)

## Abbreviations

BMI: Body Mass Index
DXA: Dual-energy X-ray Absorptiometry
HDI: Human Development Index
MEVORECH: Methodological Evaluation of Observational Research checklist
