# Supplementary material for: Body mass index and dental caries in young people: a systematic review
Source: BMC Pediatr. 2019 Apr 23;19:122. doi: 10.1186/s12887-019-1511-x (PMC6480798; doi:10.1186/s12887-019-1511-x)
Supplement: Supplementary file 1 — Reasons for exclusion of full text articles from the review. This file lists all the full text articles that were excluded from the present review along with the reason for their exclusion. (DOCX 36 kb) [file 12887_2019_1511_MOESM1_ESM.docx]

Reasons for exclusion of full text articles *(continued)*

| **Authors** | **Reasons for exclusion** |
| --- | --- |
| Abolfotouh et al., 2000 | No assessment of BMI |
| Acs et al., 1992 | No assessment of BMI & caries relationship |
| Acs et al., 1999 | No assessment of BMI & caries relationship |
| Al Ghalebi & El-Samarrai, 2012 | Insufficient data provided |
| Alvarez, 1995 | No assessment of BMI |
| Alvarez et al., 1993 | No assessment of BMI |
| Alvarez et al., 1990 | No assessment of BMI |
| Alvarez et al., 1988 | No assessment of BMI |
| Alvarez et al., 1987 | Insufficient data provided  No assessment of BMI |
| Bagri et al., 2014 | Insufficient data provided |
| Bastos et al., 2007 | No assessment of BMI |
| Batista et al., 2009 | Age range not meeting inclusion criterion |
| Bazroy et al., 2005 | No assessment of BMI & caries relationship |
| Caballero & Kakehashi, 2003 | No assessment of BMI |
| Campos et al., 2011 | No direct comparison between BMI and dental caries |
| Chakravathy et al., 2013 | Not appropriate classification of BMI |
| Chakravathy et al., 2012 | Age range not meeting inclusion criterion |
| Chang et al., 2012 | Full text not available |
| Chatterjee et al., 2012 | Age range not meeting inclusion criterion |
| Chen et al., 2004 | No assessment of BMI & caries relationship |
| Chu et al., 2013 | No assessment of caries |
| Cinar et al., 2011 | BMI not objectively measured |
| Cinar et al., 2008 | BMI not objectively measured |
| Cleaton-Jones et al., 2000 | No assessment of BMI |
| Conrey et al., 2009 | No assessment of caries |
| Correa-Faria et al., 2013 | No assessment of BMI |
| Ditmyer et al., 2011 | BMI results not reported |
| Ditmyer et al., 2008 | No assessment of BMI |
| Dye & Odjen, 2011 | Review of another study-commentary |
| Eronat & Eden, 1992 | No clear assessment of BMI, no assessment of BMI & caries relationship |
| Floyd, 2009 | Uses mean BMI for group |
| Geltman et al., 2001 | No assessment of dental caries |
| Hooley et al., 2012 | Dental caries not objectively assessed |
| Hu et al., 2004 | Conference abstract |
| Ismail et al., 2009 | No assessment of BMI |
| Ivanovic Marincovich, 1992 | No assessment of BMI |
| Jinabhai et al., 1983 | No assessment of BMI |
| Johansson et al., 1992 | No assessment of BMI |
| Justo et al., 2014 | No assessment of BMI |
| Kanchanakamol et al., 1996 | No assessment of BMI & caries relationship |
| Kay et al., 2010 | No assessment of BMI & caries relationship |
| Kramer et al., 2009 | No assessment of dental caries |
| Lalla et al., 2006 | No assessment of dental caries |
| Larsson et al., 1995 | Uses mean BMI for group |
| Larsson et al., 1997 | No assessment of BMI & caries relationship |
| Lawrence et al., 2004 | No assessment of BMI & caries relationship |
| Li et al., 1996 | No assessment of BMI |
| Liu et al., 2010 | Conference abstract |
| Mohammadi et al., 2009 | No direct assessment of BMI-caries relationship |
| Marshall et al., 2007 | Insufficient data provided |
| Masumo et al., 2014 | No assessment of BMI |
| Milaat & Grabrah, 1996 | No assessment of BMI |
| Miller et al., 1986 | No assessment of BMI |
| Miller et al., 1982 | No BMI assessment and unclear how dental caries was assessed |
| Mishu et al., 2013 | No assessment of BMI |
| Monse et al., 2013 | No assessment of BMI & caries relationship |
| Moreira et al, 2006 | No direct assessment of BMI |
| Nascimento et al., 2013 | No assessment of BMI & caries relationship |
| Ou et al., 2012 | Full text article not available in English |
| Padhy et al., 2013 | Full text not available |
| Pasdar et al., 2014 | Full text not available in English |
| Patil &Wasnik, 2009 | No assessment of BMI & caries relationship |
| Peres et al., 2005 | No assessment of BMI |
| Petti et al., 2000 | Insufficient data provided |
| Poulton et al., 2002 | Age not meeting inclusion criterion |
| Pourhashemi et al., 2007 | No assessment of BMI |
| Prashanth et al., 2011 | Insufficient data provided |
| Raducanu et al., 2011 | No assessment of BMI |
| Reyers-Perez et al., 2013 | Age range not meeting inclusion criterion |
| Scheiwe et al., 2010 | No assessment of BMI & caries relationship |
| Shahabuddin et al., 2000 | No details on caries assessment, no assessment of BMI & caries relationship |
| Shrivastava et al., 2005 | Full text not available |
| Singh et al., 2012 | Age range not meeting inclusion criterion |
| Sohn, 2009 | Commentary on another study |
| Somani et al., 2013 | Full text not available |
| Struska & Mielnik-Blaszczak, 2003 | Full text not available |
| Tambelini et al., 2010 | Age range not meeting inclusion criterion |
| Tavares et al., 2008 | No assessment of BMI |
| Thomas & Primosch, 2002 | No assessment of BMI |
| Tragler, 1981 | No assessment of BMI |
| Tuomi , 1989 | No assessment of BMI |
| Verma et al., 1989 | Full text not available |
| Walket et al., 1988 | No assessment of BMI |
| Yang et al., 2007 | No assessment of BMI & caries relationship |
