# Supplementary material for: Body mass index and dental caries in young people: a systematic review
Source: BMC Pediatr. 2019 Apr 23;19:122. doi: 10.1186/s12887-019-1511-x (PMC6480798; doi:10.1186/s12887-019-1511-x)
Supplement: Supplementary file 2 — Characteristics of studies included in the systematic review. This table presents details of all the studies that were included in the review and their citations (end of the table). The studies are grouped according to the type of relationship they identified, with some studies finding more than one pattern of relationship. (DOCX 150 kb) [file 12887_2019_1511_MOESM2_ESM.docx]

Characteristics of studies included in the systematic review *(continued)*

*Abbreviations: d: decayed, e: extracted, m: missing, e: extracted, f: filled, t: teeth, s: surfaces* (Lowercase letters: primary teeth; Uppercase letters: permanent dentition)

*Positive relationship*

| **Author, year** | **Country, City** | **Setting** | **Study design** | **Sample size and gender distribution (n, %)** | **Age group (years)** | **HDI category** | **Definition of weight status** | **Measure of dental caries** | **Results as reported by the authors** |
| --- | --- | --- | --- | --- | --- | --- | --- | --- | --- |
| Alm et al., 2008 | Sweden, Mmunicipality of Jönköping | Schools & Public dental service clinics | CS of a longitudinal cohort | 402 at age 15 years  Boys: 206 (51.2)  Girls: 196 (48.8) | 15 years | Very high | BMI - using the international classification system  for childhood obesity (isoBMI) recommended  by the IOTF cut-off values.  (i) low normal weight (isoBMI < 25); (ii) overweight (isoBMI 25–29.9); and (iii) obesity (isoBMI ≥30). | D_ia_-initial caries  D_ma -_Manifest caries  D_i+m_Fa,-total approximal caries prevalence and fillings | Adolescents with isoBMI ≥ 25 (n= 64) had an approximal caries prevalence that was a mean  of 1.6 times higher than those with isoBMI < 25 (n= 338) (4.64 vs. 2.94; p = 0.014). |
| Alm et al., 2011 | Sweden, Municipality of Jönköping | Child welfare centers, schools & Public dental service clinics | CS of a longitudinal cohort | 525 at age 3 years  506 at age 6 years  402 at age 15 years  Gender details not provided | 1-15 years | Very high | BMI - using the international classification system for childhood obesity (isoBMI) recommended by the IOTF cut-off  values. (i) low normal weight (isoBMI < 25); (ii) overweight (isoBMI 25–29.9); and (iii) obesity (isoBMI ≥30). | At age 3 and 6 years: defs total and defs manifest  At age 15 years:  D_i_a  D_m_a  D_i+m_Fa. | At 6 years of age, the odds (OR) of having caries among obese children are 2.5 times higher than the odds for caries among six-year-old children of normal weight (p = 0.04). Adolescents (15 years) who were overweight/obese had more than twice as many approximal carious lesions and fillings compared with low-normal weight individuals (p<0.05). In the low-normal weight group, 80% were free from approximal manifest carious lesions and fillings compared with 59% in the obese group and the difference was statistically significant (Fisher's exact text, p=0.04 and OR=2.8; 95% CI: 1.0-7.7) |
| Bagherian & Sadeghi 2013 | Iran, Rafsanjan | Private and state-funded preschools | CS | 400  Boys: 211 (52.8)  Girls: 189 (47.2) | 30-70 months | High | BMI-for-age  Using standardized centiles derived from the first National Health and Nutrition Examination Study, 1971 to 1974  Underweight was defined as BMI-for-age <5 th percentile, Normal-weight 5th percentile < BMI-for-age < 85th percentile,  At risk of overweight 85th percentile < BMI-for-age < 95th percentile, and  Overweight BMI-for-age >95 th percentile. | defs | The mean and SD of defs index was 8.37 ± 11.2. In the underweight, normal-weight, at risk of overweight and overweight groups, these values were 4.89 ± 10.8, 8.84 ± 11.8, 8.68 ± 10.6, and 10.39 ± 10.2, respectively.  Multiple regression analysis revealed a statistically a significant direct association between BMI-for-age and defs index (P = 0.001) after adjusting for gender and age. |
| **Author, year** | **Country, City** | **Setting** | **Study design** | **Sample size and gender distribution (n, %)** | **Age group (years)** | **HDI category** | **Definition of weight status** | **Measure of dental caries** | **Results as reported by the authors** |
| Bener et al., 2013 | Qatar | Primary Health Care Centers (PHCs) | CS | 1249  Boys:772 (61.8)  Girls: 477 (38.2) | 6–15 years | Very high | BMI for age- CDC  Normal: <85^th^ percentile  Overweight: 85-95^th^ percentile  Obese:>95^th^ percentile | DMFT | Having a BMI greater than the 95th percentile versus less than the 85th percentile (adjusted OR: 2.12; 95% CI: 1.17–3.84) was associated independently with the risk of dental caries among children in Qatar. |
| Bhoomika et al., 2013 | India, Mathura city | Department of Pedodontics and Preventive Dentistry (K D Dental College and Hospital)and schools | CC | 200-  100 caries free children (50 boys and 50 girls)  and 100 children (50 boys and 50 girls) affected with S-ECC | 3-6 years | Medium | BMI-for -age percentiles utilizing age and gender specific CDC BMI percentiles  1) underweight (<5thpercentile), 2) normal weight (fifth to 84th percentile),  3) at risk for overweight (>85th to 95th percentile) and  4) overweight (>95^th^ percentile) | defs | For the caries free children 51% are underweight, 45% are normal,  1% at risk of overweight and 3% were observed to be overweight. In  the S-ECC affected children 45% are underweight, 41% normal, 4%  at risk of overweight and 10% were observed to be overweight. The mean BMI of S-ECC children is more when compared to the caries free children which was found to be statistically significant at p<0.05 |
| Cantekin et al.,2012 | Turkey, Erzurum | Department of Pediatric  Dentistry, Faculty of Dentistry, Ataturk University. | CS | 224 (boys) | 12 years | High | BMI- The BMI was categorized into four Groups according to the International Obesity Task Force cut off values.  low weight (BMI-1),  normal weight (BMI-2), overweight (BMI-3),  and obesity (BMI-4) | DMFT | Respective mean DMFT values for BMI-1, BMI-2, BMI-3, and BMI-4 were 1.44, 1.47, 1.81, and 2.33, respectively. There were significant differences between BMI-1 and BMI-4 (PZ0.019, r2 Z 0.73) and between BMI-2 and BMI-4 (P Z 0.022, r2Z 0.72) values. |
| Dos Santos Junior et al., 2014 | Brazil,  Cabo de Santo Agostinho, southeastern part of Pernambuco | Municipal kindergartens | CS | 320  Unclear if sample included children of both genders | 3-4 years | High | BMI-for -age percentiles utilizing age and gender specific CDC BMI percentiles  1) underweight (<5thpercentile), 2) normal weight (fifth to 84th percentile),  3) at risk for overweight (>85th to 95th percentile) and  4) overweight (>95th percentile) | ECC | Obesity was related to the increase of ECC (p<0.001).  Univariate PR 95% CI: 3,40 (1.89 a 6.11)  Adjusted PR 95% CI: 6,24 (3.06 a 12.72) |
| Fadel et al., 2014 | Goteborg, Sweden | Obesity clinic | CC | 55  29 boys  26 girls | 16±2 years | Very high | BMI-Children were classified as obese based on the IOTF classification. | DFT, DFS,DS, FS | Compared with controls, adolescents with obesity had, more decayed  tooth surfaces (3.4±6.6 vs. 0.8±1.1, p<0.05) |

| **Author, year** | **Country, City** | **Setting** | **Study design** | **Sample size and gender distribution (n, %)** | **Age group (years)** | **HDI category** | **Definition of weight status** | **Measure of dental caries** | **Results as reported by the authors** |  |
| --- | --- | --- | --- | --- | --- | --- | --- | --- | --- | --- |
| Gerdin et al., 2008 | Sweden, County of Ostergotland | Child welfare centres,school & public dental service clinics | CS longitudinal cohort | 2303  Gender distribution not provided | 4-12 years | Very high | BMI - using the international classification system  for childhood obesity (isoBMI) recommended  by the IOTF cut-off  values.  Overweight (>25 kg ⁄m2 at 18 years) and  Obesity  (>30 kg ⁄m2 at 18 years) | deft (6 years)  DFT (10 &  12 years),  DFSa | Obese, but not overweight, children had more caries affected teeth than  non-obese, and BMI had an independent, though weak, effect on caries. Children  who were obese at 4 years of age had 1.1  caries affected teeth when they were 12 years old compared with 0.7 affected teeth (P = 0.027) in those who had normal weight at age 4. |  |
| Hilgers et al., 2006 | Louisville Metropolitan area, US | Dental school | CS | 178  85 boys  93 girls | 8-11 years | Very high | BMI-international classification system recommended by IOTF.  Categories used include normal weight, overweight and obese | Radiographs  c-avg, C-avg | Elevated body mass index is associated with an increased incidence of permanent molar interproximal caries.  **C-avg ±SD**  Low BMI: 0.08 ±0.06  Normal BMI: 0.19± 0.05  High BMI: 0.51 ± 0.09 |  |
| Honne et al., 2012 | India, Udupi District | Private and government schools | CS | 463  Boys:252 (54.4)  Girls: 211 (45.6) | 13-15 years | Medium | BMI- according  to the IOTF cut-off values  Low- normal weight (BMI < 25), Overweight (25–29.9) and Obesity (BMI ≥30) | DMFT | Caries experience had a significant association with overweight /obese status(OR = 3.68, CI = 1.79–7.56) P<0.05. |  |
| Loyola-Rodriguez et al., 2011 | Mexico | Oral medicine clinic and laboratory | CC | 100  50 OB-IR  50 without OB-IR  Unclear if sample included both genders | 12-18 years | High | BMI- 2000 CDC growth charts for the US.  Obesity: BMI≥ 95^th^ percentile  Overweight: BMI≥ 85^th^ percentile and < 95^th^ percentile  Normal weight: BMI <85^th^ percentile | DMFT | DMFT index was 3.02 for healthy and for OB-IR adolescents was 4.78, showed a significant statistical difference (p < 0.05). |  |
| Modeer et al., 2010 | Stockholm ,Sweden | University hospital- Division of Pediatric Dentistry | CC | 130  65 obese  65 normal weight  Gender distribution not provided | Obese mean age: 14.5 years  Healthy: 14.2 years | Very high | BMI IOTF classification and BMI adjusted for age and sex (BMI-sds)  Obese: ISO-BMI >30  Normal weight: ISO-BMI <25 | DMFT/S | The obese subjects exhibited higher number of decayed surfaces (DS), 0.7 vs. 0.1 (P = 0.008). In a multivariate logistic regression model BMI-sds was significantly associated with DS (DS >0) (P = 0.002; OR 1.31). |  |
| **Author, year** | **Country, City** | **Setting** | **Study design** | **Sample size and gender distribution (n, %)** | **Age group (years)** | **HDI category** | **Definition of weight status** | **Measure of dental caries** | **Results as reported by the authors** |  |
| Powell et al., 2013 | USA, Carolina | UNC Children’s Hospital & Department of  Pediatric Dentistry, UNC School of Dentistry | CS-Retrospective cohort | 215  Boys: 119 (55)  Girls: 96 (45) | 3-5 years | Very high | BMI percentiles-  P_25_  Median  P_75_ | dmft | BMI percentile was a statistically significant explanatory variable for dmft after controlling for race, gender, and age (P<.001). When BMI percentile increased by 10 units, the dmft score increased by an estimated 1.1 after adjusting for the other covariates. |  |
| Qadri et al., 2015 | North-East Germany, District of Eastern Pomerania and Greifswald  city | Primary schools | CS- prospective cohort study | 694  Boys: 52%  Girls: 48% | 9-12 years | Very high | BMI - using the international classification system  for childhood obesity (isoBMI) recommended  by the IOTF cut-off  values.  Low normal weight: isoBMI ≤ 25  Overweight: isoBMI >25–29.9 Obesity: isoBMI≥30. | DMFT | Low-normal weight children had a lower mean DMFT (0.56) than did overweight/obese children (0.70). In addition, a border-line significant association was found between overweight/obese children and caries  increment (P = 0.055). | |
| Reisfnider et al., 2014 | US | Clinics | CS | 104  59 Boys  45 girls | 1-20 years | Very high | BMI-Classification system not specified | ECC | Children with a greater  degree of caries experience tended to have higher BMI's than those with less caries, r (df =100) = .20, p < 0.05. | |
| Sakeenabi et al., 2012 | India, Davangere city, Karnataka state | Schools | CS | 1550  Boys: 798 (51.5)  Girls: 752 (48.5) | 6 and 13 years | Medium | BMI –for- age percentiles  utilizing age and gender specific CDC BMI percentiles  Underweight: less than 5^th^ percentile;  Normal weight: 5th percentile  to less than 85th percentile;  At risk of overweight: 85th to less than 95th percentile;  Overweight: equal to or greater than the 95^th^ percentile | DMFT and  dmft | In 6-year olds, obese children were 3.6 (OR = 3.6; 95% CI = 2.5–4.32) times more likely to have dental caries  than subjects who were not obese Similarly, in 13-year-olds, obese children were 1.8 times more likely to have dental caries (adjusted OR = 1.8;  95% CI = 1.1–2.8) than non-obese children. | |
| Shahraki et al., 2013 | Iran, Zahedan | Primary schools | CS | 1213  Boys: 543 (44.8)  Girls: 670 (55.2) | 6-11 years | High | BMI-for-age  Underweight: BMI-for-age < 5th percentile,  Normal: 5th percentile < BMI-for-age < 85th percentile,  At risk of overweight” : 85th percentile < BMI-for-age < 95th per­centile, and  Obese: BMI-for-age > 95th percentile | DMFT & DFT | In the low weight, normal weight, overweight, and obese groups, the mean ± SD values for DFT were: 0.63 ± 1.1, 0.88 ± 1.36, 1.16 ± 1.33, and 0.87 ± 1.31, respectively. There was a significant association between BMI and DFT (P = 0.005). | |
| **Author, year** | **Country, City** | **Setting** | **Study design** | **Sample size and gender distribution (n, %)** | **Age group (years)** | **HDI category** | **Definition of weight status** | **Measure of dental caries** | **Results as reported by the authors** | |
| Sharma & Hedge, 2009 | India, Mangalore city | Department of Pedodontics and Preventive Children Dentistry, AB Shetty Memorial Institute of Dental Sciences | CS | 500  Boys: 255 (51)  Girls: 245 (49) | 8-12 years | Medium | BMI –for- age percentiles  utilizing age and gender specific CDC BMI percentiles (2000)  Underweight: <5^th^ percentile  Normal: 5^th^ -85^th^ percentile  Risk overweight:85^th^-95^th^  Obese:>95^th^ | DMFS/dfs  dmfs/DMFS | Children with obesity and overweight had increased prevalence of dental caries in both primary and permanent dentition compared to normal weight children, which was statistically significant.  **Mean DFMS:**  Underweight: 3.11  Normal  weight: 1.58  Overweight: 2.48  Obese: 2.85  **Mean dfs:**  Underweight: 2.00  Normal weight: 2.14  Overweight: 4.79  Obese: 3.25 | |
| Subramaniam & Singh, 2011 | India, Bangalore City, Karnataka | Schools | CS | 2033 children  Boys: 1021 (50.2) Girls: 1012 (49.8) | 6-15 years | Medium | BMI –for- age percentiles  utilizing age and gender specific CDC BMI percentiles  Underweight: BMI-for-age < 5^th^ centile  Normal: BMI-for-age ≥5^th^ <85^th^ centile  Risk of overweight: BMI-for-age ≥85^th^ < 95^th^ centile  Overweight: BMI-for-age >95^th^ | deft, DMFT | A significantly higher mean DMFT score was observed in children at risk of overweight and overweight children.  **DMFT mean ±SD**  Underweight: 062±1.17  Normal:0.72±1.37  Risk of overweight:1.36±1.89  Overweight:1.26±1.38 | |
| Thippeswamy et al., 2011 | India, Udupi district | Private and government schools | CS | 463  Boys: 252 (54.4)  Girls: 211 (45.6) | 13-15 years | Medium | BMI - using the international classification system  for childhood obesity (isoBMI) recommended  by the IOTF cut-off  values.  (i) low normal weight (isoBMI < 25); (ii) overweight  (isoBMI 25–29.9); and (iii) obesity (isoBMI  ≥30). | DMFT | The obese group of children had more caries than the overweight and low normal weight children.  Correlation analysis showed significant positive relation with BMI, decayed teeth (DT) [r = 0.254, p < 0.001] and DMFT (r = 0.242, p < 0.001). | |
| Trikaliotis et al., 2011 | Greece, Thessaloniki | Municipal day care centres | CS | 361 children  Boys: 183 (50.7)  Girls: 178 (49.3) | 3-5.5 years | Very high | BMI-for-age-IOTF  Underweight: <18.5kg/m² at age 18,  Overweight: >25kg/m² at age 18  Obesity >30kg/m² at age 18 | dmfs | Mean dmfs values for each BMI category were: 1.02 (SD±2.41) for the  underweight (n=44), 0.74 (SD±2.24) for the normal weight (n=281), 1.88 (SD±4.28) for the overweight (n=26) and  0.80 (SD±2.53) for the obese (n=10). Overweight children were found to show statistically significant differences in  dmfs values compared with both children of normal weight (p<0.001) and those underweight (p=0.015). | |
| **Author, year** | **Country, City** | **Setting** | **Study design** | **Sample size and gender distribution (n, %)** | **Age group (years)** | **HDI category** | **Definition of weight status** | **Measure of dental caries** | **Results as reported by the authors** | |
| Vazquez-Nava et al., 2010 | Mexico, Area of Tampico-Madero-Altamira | Nursery schools | A CS study based on a cohort of 1,160 children | 1,160  Boys: 582 (50.2)  Girls: 578 (49.8) | 4-5 years | High | BMI-for -age percentiles utilizing age and gender specific CDC BMI percentiles  Normal weight: 5-85th percentile  At-risk overweight: ≥85^th^ and <95th percentile;  Overweight:≥95th | deft  defs | **Mean (SD) deft:**  Total sample = 1.08 (2.33)  Normal weight= 0.70 (1.94)  At -risk overweight =1.50 (2.57)  Overweight = 1.51 (2.71)  **Mean (SD) defs:**  Total sample = 1.43 (3.28)  Normal weight = 0.93 (2.64)  At-risk overweight = 1.95 (3.49)  Overweight= 2.04 (3.97)  When adjusted for covariates, the  logistic regression model showed that there was a significant association between at-risk overweight children (P < 0.001), overweight children (P < 0.001), and caries in the primary dentition. | |
| Willerhausen et al., 2007 | Germany | Dental hospital | CS | 1290  Boys: 50.2%  Girls: 49.8% | 6-11 years | \Very high | BMI- Calculation based on special tables from the obesity consortium for children and adolescents.  Low weight (BMI<20),  Normal weight (BMI: 20-25),  High weight (BMI: 25-30), Obesity (BMI>30) | DF-T  and df-t | **Mean df-t (±SD)**  Underweight= 1.43 (±2.02)  Normal weight= 1.82 (±2.41)  High weight= 2.3 (±2.75)  Obese= 2.21 (±2.8)  **Mn DF-T (±SD)**  Underweight= .38(±1.28)  Normal weight= 0.53 (±1.2)  High weight= 0.85(±1.4)  Obese= 0.82 (±1.3)  A significant association between high weight and caries frequency in the first dentition (p= 0.0067) and in the permanent dentition (p=0.0002) could be observed. | |
| Willershausen et al., 2004 | Germany, Mainz | Elementary schools | CS | 842  Boys:428 (50.8)  Girls: 414 (49.2) | 6 -12 years (results)  6-11 (methods) | Very high | BMI- Calculation based on special tables from the obesity consortium for children and adolescents (Arbeitsgemeinschaft Adipositasin Kindes-und Jugendalter 2002)].  Low weight (BMI<20),  Normal weight (BMI: 20-25),  High weight (BMI: 25-30), Obesity (BMI>30) | DF-T- (df-t) | The caries prevalence (DF-T-, df-t-values) showed a significant association to weight (Fisher-Test, p = 0.022 for df-t-distribution and p = 0.011 for DF-T-distributions). Children with normal weight were found to have average df-t-values of 2.09 (DF-T: 0.57), overweight children an average df-t-value of 2.48 (DF-T-value: 0.91), and obese children showed 3.3 (DF-T.value:0.88). | |
| **Author, year** | **Country, City** | **Setting** | **Study design** | **Sample size and gender distribution (n, %)** | **Age group (years)** | **HDI category** | **Definition of weight status** | **Measure of dental caries** | **Results as reported by the authors** | |
| Willershausen et al., 2007 | Germany, Mainz | Elementary schools | CS | 2071  Boys: 998 (48.2)  Girls: 1073 (51.8) | 6-10 years | \Very high | BMI-. Calculation based on special tables from the obesity consortium for children and adolescents | DF-T , df-t | **Mean df-t +DF-T**  Underweight= 1.67  Normal weight = 2.15  Overweight = 2.64  Obese= 2.7  High BMI was linked to a high number of caries lesions (df-t + DF-T  values; p = 0.0021). | |

*Negative relationship*

| **Author, year** | **Country, City** | **Setting** | **Study design** | **Sample size and gender distribution (n, %)** | **Age group (years)** | **HDI category** | **Definition of weight status** | **Measure of dental caries** | **Results as reported by the authors** |
| --- | --- | --- | --- | --- | --- | --- | --- | --- | --- |
| Alkarimi et al., 2014 | Saudi Arabia, Jeddah | Military primary schools | CS | 417  Boys: 175 (42)  Girls: 242 (58) | 6 - 8 years | Very high | BMI-for-age z scores  WHO Reference 2007 (ages 5 to19)  A cut off of <-2SDs was used to report underweight and stunting, whereas a cutoff of >+SDs was used to report obesity. | dmft | There was an inverse linear relationship between  caries status and children’s HAZ, WAZ, and BAZ and significantly lower  anthropometric outcomes for children at each consecutive group with higher levels of caries. The adjusted mean BAZ values for caries categories (dmft ≤2, 3-6 and ≥7) were 0.40,  0.12, and 0.21, respectively (P = 0.001). |
| Benzian et al., 2011 | Philippines | Schools | CS | **1951**  Boys: 949 (48.6)  Girls:1002 (51.4) | 11-13 years | Medium | BMI-for-age- The children were grouped in 3 categories of BMI with age and sex related cut-off points according to the criteria of WHO (de Onis et al., 2007), CDC (Kuczmarkai et al., 2000), Philippines NHANS I (Department of Education, Health and Nutrition Center 1991) and Cole and colleagues (2007). For further analyses, the 3 BMI categories according to the Philippines NHANS I criteria were used. | DMFT/dmft and PUFA/pufa | The regression coefficient between BMI and caries was highly significant (p < 0.001). Children with  PUFA + pufa > 0 as compared to those without odontogenic infections had an increased risk of a  below normal BMI (OR: 1.47; 95% CI: 1.19-1.80). |
| Cameron et al., 2006 | Scotland, UK | Glasgow Dental Hospital | CS | 165  52%girls  48%boys | 3-11 years | Very high | BMI –UK 1990 growth reference adjusting for age and gender. | Dmft,  DMF | Children with the worst decay were also significantly thinner (p=0.019).  **Dmft 1-7 8-20**  BMI SD 0.18 -0.42 |
| Creske et al., 2013 | California, US | Elementary schools | CS | 177  68 boys (38.4%)  109 females (61.6%) | 3^rd^ grade students from elementary school | Very high | BMI –  The BMI was calculated using the tool provided by  the Center for Disease Control and Prevention | DMFT, | Children from the obese category were less likely  to have dental caries (OR=0.68, 95% CI (0.48,  0.98)) than children in the normal weight category,  and this was statistically significant (p=0.04). |
| **Author, year** | **Country, City** | **Setting** | **Study design** | **Sample size and gender distribution (n, %)** | **Age group (years)** | **HDI category** | **Definition of weight status** | **Measure of dental caries** | **Results as reported by the authors** |
| Frazao et al., 2014 | Brazil, Urban area  of a small town within the western Brazilian Amazon | Schools & Households | CS survey nested in a population-based cohort study | 203  Boys:95 (46.8)  Girls: 108 (53.2) | 7-9 years | High | BMI-for-age z scores  WHO Reference 2007 (ages 5 to19)  Children were classified in quintiles. | dmft/DMFT index | **Untreated decayed deciduous and permanent teeth: mean (SD)**  Lower quintile 4.53 (3.70)  Second quintile 3.32 (2.90)  Third quintile 3.82 (3.29)  Fourth quintile 3.66 (3.49)  Upper quintile 2.84 (3.20)  Reduced caries experience was observed in children from the upper quintile of Z- scores for BMI. |
| Freitas et al., 2014 | Brazil, State of Sao Paulo, | Public schools | CS | 202  Group O -overweight/obese: 101 (45 boys & 45 girls)  Group N - normal: 101 (45 boys & 45 girls) | 12 years | High | BMI-for-age z scores  WHO Reference 2007 (ages 5 to19)  Overweight/obese: BMI≥85^th^ percentile  Normal weight: BMI ≥ 3^rd^ <85^th^ percentile | DMFT & SiC | In group O, 42.57% of the adolescents were caries free compared with 28.71% in group N. The mean DMFT score was 1.67 (D = 0.65; M = 0.00; F = 1.02) in group O and 2.12 (D = 1.03; M = 0.02; F = 1.07) in group N; the SiC score was 3.85 in group O and 4.26 in group N. A significant relationship was observed between BMI-for-age and SiC (OR = 0.649; 95% CI: 1.093–4.010; P = 0.024). |
| Goodson et al., 2013 | Kuwait | Primary schools | CS | 8275  Boys: 3170 (38.3)  Girls: 5105 (61.7) | 11.36 ± 0.1 years | Very high | BMI-for-age z scores  WHO Reference 2007 (ages 5 to19)  Obese: Z-score > than 2 SD,  Overweight: Z-score >1 SD, Normal healthy  Weight: Z-score between1 and -2 SD.  Underweight: Z-score  <-2 SD | Number of teeth with  fillings and visible unfilled decay | The percentage of decayed or filled teeth decreased from 15.61% (n=193) in underweight children, to 13.03% (n=4,094) in normal healthy weight children, to 9.73% (n=1,786) in overweight children to 7.87% (n=2,202) in obese children.  Differences between all groups were statistically significant. |
| **Author, year** | **Country, City** | **Setting** | **Study design** | **Sample size and gender distribution (n, %)** | **Age group (years)** | **HDI category** | **Definition of weight status** | **Measure of dental caries** | **Results as reported by the authors** |
| Jahani et al., 2013 | Southeast Iran, Kerman | Primary schools | CS | 906  Boys: 487 (53.8)  Girls: 419 (46.2) | 12 years | High | BMI- Standardized percentile curves of BMI in Iran  Underweight: under the 5th percentile curve,  Normal: between the 5th and 85th percentile,  At risk of overweight: higher than the 85th and lower than the 95th percentile and  Overweight as higher or equal to the 95th percentile. | dmft/DMFT | The dmft/DMFT adjusted rate for over- weight pupils was 0.86 (95% CI: 0.74 - 0.98) times lower than those with normal weight. |
| Kopycka-Kedzierawski et al., 2008 | USA | Mobile offices and households | CS | A total of 10 180 participants  **2-5yrs**:  Boys: 2048/Girls: 2119  **6-11yrs:**  Boys:1502/Girls: 1367  **12-18yrs:**  Boys: 1304/Girls: 1473 | 2-18 years | Very high | BMI-for -age percentiles utilizing age and gender specific CDC BMI percentiles  Overweight: at or above the 95th percentile  At risk for overweight: at or  above the 85th percentile and less than 95th  percentile  Normal weight: less than 85th percentile | Dfs (2-11 years) and DMFS (6-18 years) | **Prevalence of caries (SE)**  **6-11years old (Primary dentition)**  Normal weight= 51.4 (1.6)  At risk = 45.5 (4.6)  Overweight= 40.6 (4.7)  **6-11yrs old (Permanent dentition)**  Normal weight= 26.5 (1.7)  At risk = 29.9 (4)  Overweight= 17.6 (2.9)  For children 6–11 years of age (NHANES III), at risk for overweight and overweight children were less likely to have caries experience in the primary dentition than normal weight children; overweight children were less likely to have caries experience in the permanent dentition than normal weight children.  **Prevalence of caries (SE)**  **12-18 years old (Permanent dentition)**  Normal weight= 67.2 (2.2)  At risk =67.8 (4.7)  Overweight= 57.7 (4.6)  For children 12–18 years of age (NHANES III), overweight children were less likely to have caries experience in the permanent dentition than normal weight children. |
| **Author, year** | **Country, City** | **Setting** | **Study design** | **Sample size and gender distribution (n, %)** | **Age group (years)** | **HDI category** | **Definition of weight status** | **Measure of dental caries** | **Results as reported by the authors** |
| Koskal et al., 2011 | Turkey, Ankara | Primary schools | CS | 245  Boys: 50.2%  Girls: 49.8% | 5-9 years | High | BMI-for-age z scores  WHO Reference 2007 (ages 5 to19)  Low weight, normal weight and overweight-obese,  in accordance with the cut-off points of <−1SD, ≥−1SD — +1SD and ≥+1SD z-scores, respectively. | DMFT &DMFS | BMI was found to be negatively correlated with dmft indices (r:−0.139, p: 0.030) |
| Narksawat et al., 2009 | Thailand | Primary schools | CS | 862  Boys:411 (47.7)  Girls: 451 (52.3) | 12 - 14 years | High | BMI centiles- Other classification  according to the Division of Nutrition, Thai  Ministry of Public Health standard manual  using weight for height in Thai children. Median with standard deviation (SD) was used as the cutoff point for nutritional status.  Very thin: less than median minus 2 SD;  Thin: median minus 2 SD to median minus 1.5 SD;  Normal: median minus  1.5 SD to median plus 1.5 SD; Overweight: median plus 1.5 SD to plus 2 SD; and  Obese: more than median plus 2 SD | DMFT | The results from multiple logistic regression analysis showed normal weight and thin schoolchildren were more likely to have a DMFT of at least 1 by 1.94 times (OR = 1.94; 95%CI = 1.25-3.00, p = 0.004) and 2.22 times (OR = 2.22; 95%CI = 1.20-4.09, p =0.001), respectively, compared to overweight and obese children. |
| Ngoenwiwatkul & Leela-adisorn, 2009 | Thailand | Primary schools | CS | 212  Boys: 117 (55.2)  Girls: 95 (44.8) | 6-7 years | High | BMI-for -age percentiles utilizing age and gender specific CDC (2007) BMI percentiles  with some modification  Underweight: BMI-for-age  <5th percentile and  “At risk” of being underweight: BMI-for-age from 5th to 15th percentile.  In the analysis phase, no one had BMI-for-age  < 5th percentile categories, thus the term “underweight” was used to refer to a group with <15th percentile of BMI-for-age. | dmfs &dmft | Multiple logistic regression showed that each extra carious surface (dmfs)  increased the odds of being at risk for underweight (5th < BMI-for-age < 15th) by 3.1% after adjusting for gender and dental visits. |
| **Author, year** | **Country, City** | **Setting** | **Study design** | **Sample size and gender distribution (n, %)** | **Age group (years)** | **HDI category** | **Definition of weight status** | **Measure of dental caries** | **Results as reported by the authors** |
| Norberg et al., 2012 | Southern Sweden. Ska°ne county | Child Health Care Centers | CS | 920  Boys: 466 (50.7)  Girls: (49.3) | 5 years | Very high | BMI - using the international classification system  for childhood obesity (isoBMI) recommended by the IOTF cut-off values and also statistical  cutoffs for the groups normal weight, low weight, and underweight based on national epidemiological data on BMI in 5-year-olds in Sweden.  Overweight: ISO BMI ≥ 25  Obesity: ISO BMI ≥ 30  This study used the international  definition of overweight (ISO BMI ≥ 25) to define the combination of groups H (high weight) and O (obese), and the international definition of obesity (ISO BMI _ 30) to define group O . Group H was defined as ISO BMI ≥25 and < 30. | deft and dt | **Deft mean (SD)**  U=2.00  L=1.27  N=0.65  H=0.75  Children in groups U and L had higher deft scores  than children in group N (P = 0.010 and  P = 0.025,respectively). |
| Oliveira et al., 2008 | Brazil, Diadema | Health centres | CS | 1018  Boys: 519 (51)  Girls: 499 (49) | 12-59 months | High | BMI-for-age z scores  WHO Child Growth Standards 2006  At risk underweight: z score <-2  Normal: -2≤ z score ≤+2  At risk overweight: z score>+2 | dmfs | Children with low BAZ-scores were more likely to  have experienced dental caries (OR = 3.20, P = 0.011); |
| Sanchez-Perez et al., 2010 | Mexico city | Public elementary school | Longitudinal | 110 (88 follow up)  Unclear if both genders | 7-11 follow up | High | BMI -CDC 2000  Overweight: BMI> 95^th^ percentile  Risk of overweight: BMI≥ 85^th^ percentile and <95^th^ percentile  Normal: BMI≥ 50^th^ percentile and <85^th^ percentile  Thin: <50^th^ percentile and >5^th^ percentile  Underweight: <5^th^ percentile | DMFT, DMFS  Dmft, dmfs | A lower dmfs index was detected in the overweight children, compared with children with a lower BMI (p<0.001).  **Dmfs coefficient**  Normal: -0.99  Risk of overweight: -0.12  Overweight: -2.01 |
| Shakya et al., 2013 | India, Mangalore city | Not reported | CS | 248  Gender distribution not provided | 6,10 and 12 years | Medium | BMI –for- age percentiles  utilizing age and gender specific CDC BMI percentiles  Underweight: less than 5^th^ percentile;  Normal weight: 5th percentile  to less than 85th percentile;  At risk of overweight: 85th to less than 95th percentile;  Overweight: equal to or greater than the 95^th^ percentile.  . | dft/DFT | The Pearson correlation test result suggested children with less BMI score tend to  have more caries affected teeth than children  with normal BMI.  Body Mass Index score and dft score (r=-0.016)  For DFT score of permanent dentition of age groups 10  and 12 r= (-0.108 and -0.033 respectively). |
| **Author, year** | **Country, City** | **Setting** | **Study design** | **Sample size and gender distribution (n, %)** | **Age group (years)** | **HDI category** | **Definition of weight status** | **Measure of dental caries** | **Results as reported by the authors** |
| Sharma & Hedge, 2009 | India, Mangalore city | Department of Pedodontics and Preventive Children Dentistry, AB Shetty Memorial Institute of Dental Sciences | CS | 500  Boys: 255 (51)  Girls: 245 (49) | 8-12 years | Medium | BMI –for- age percentiles  utilizing age and gender specific CDC BMI percentiles (2000)  Underweight: <5^th^ percentile  Normal: 5^th^ -85^th^ percentile  Risk overweight:85^th^-95^th^  Obese:>95^th^ | DMFS/dfs  dmfs/DMFS | Underweight children had  significantly higher DMFS rates(but not dfs) than normal weight, overweight and obese children.  **Mean DFMS:**  Underweight: 3.11  Normal  weight: 1.58  Overweight: 2.48  Obese: 2.85  Mean dfs:  Underweight: 2.00  Normal weight: 2.14  Overweight: 4.79  Obese: 3.25 |
| Subramaniam & Singh, 2011 | India, Bangalore City, Karnataka | Schools | CS | 2033 children  Boys: 1021 (50.2) Girls: 1012 (49.8) | 6-15 years | Medium | BMI –for- age percentiles  utilizing age and gender specific CDC BMI percentiles  Underweight: BMI-for-age < 5^th^ centile  Normal: BMI-for-age ≥5^th^ <85^th^ centile  Risk of overweight: BMI-for-age ≥85^th^ < 95^th^ centile  Overweight: BMI-for-age >95^th^ | deft, DMFT | The mean deft score was significantly higher in underweight children.  **deft mean ±SD**  Underweight: 1.29±1.89  Normal:0.89±1.47  Risk of overweigh0.82:±1.21  Overweight:1.21±1.55 |
| Werner et al., 2012 | USA, North Carolina | Undergraduate pédiatrie dentistry elinie at the School of Dentistry | CS& retrospective review of a cohort | 230  Boys: 116 (50.4)  Girls: 114 (49.6) | 6- 9 years | Very high | BMI-for -age percentiles utilizing age and gender specific CDC BMI percentiles (for 2-19 years). | DT, dt | **Primary teeth caries N(%)**  Underweight/normal:81(51)  Overweight:10(30)  Obese: 10(34)  A smaller proportion of OB and OW children  presented with primary tooth caries than UW/H children (P=0.04). |

*No relationship*

| **Author, year** | **Country, City** | | **Setting** | **Study design** | **Sample size and gender distribution (n, %)** | **Age group (years)** | **HDI category** | **Definition of weight status** | **Measure of dental caries** | | **Results as reported by the authors** | |
| --- | --- | --- | --- | --- | --- | --- | --- | --- | --- | --- | --- | --- |
| Alm et al., 2011 | Sweden, Municipality of Jönköping | | Child welfare centers, schools & Public dental service clinics | CS of a longitudinal cohort | 525 at age 3 years  506 at age 6 years  402 at age 15 years  Gender details not provided | 1-15 years | Very high | BMI - using the international classification system  for childhood obesity (isoBMI) recommended  by the IOTF cut-off values.  (i) low normal weight (isoBMI < 25); (ii) overweight (isoBMI 25–29.9); and (iii) obesity (isoBMI ≥30). | At age 3 and 6 years: defs total and defs manifest  At age 15 years:  D_i_a  D_m_a  D_i+m_Fa. | | At 3 years of age, no association between overweight/obesity and caries was found. In the low-normal weight group (n=449), 86% were free from manifest carious lesions and filings compared with 93% in the obese group (n=15)  **Defs total-Mean (SD)**  isoBMI < 25: 1.75(5.11)  isoBMI ≥25: 1.01(3.23)  isoBMI 25–29.9:1.02(3.16)  isoBMI ≥30:1.00(3.61) | |
| Alves et al., 2013 | Southern Brazil, Porto Alegre | | Schools | CS | 1528  Boys:770 (50.4)  Girls:758 (49.6) | 12 years | High | BMI-for-age z scores  WHO Reference 2007 (ages 5 to19)  Normal weight (BMI-for-age Z-score _ +1 SD),  Overweight (BMI-for-age Z-score > +1 SD to _ +2 SD),  Obese (BMI-for-age Z-score > +2 SD). | DMFT  The presence of incipient caries  lesions  was also recorded. | | No significant differences in caries experience or extent  were observed among BMI groups. After adjusting for important cofactors,  weight status was not associated with caries prevalence (overweight, PR = 0.99; 95% CI = 0.89–1.10; obese, PR = 1.00; 95% CI = 0.87–1.16) or caries extent (overweight, RR = 0.91; 95% CI = 0.74–1.12; obese, RR = 0.86; 95% CI = 0.72–1.04). | |
| Chen, 1998 | Taiwan, Taipei city, | | Original article in Chinese | CS | 5133  Boys:2822 (55)  Girls:2311 (45) | 3 years | High | BMI-for-age  Using standardized centiles derived from the first National Health and Nutrition Examination Study, 1971 to 1974 (Hammer et al., 1991).  <5^th^ percentile: very low BMI  5^th^ - 25^th^ percentile: low BMI  25^th^-75^th^ percentile: medium BMI  75^th^-95^th^ percentile: high BMI  >95^th^ percentile: obese | dft | | There were no significant differences in the dft score of carious children among different BMI groups.  **dft score**  BMI >95% tile:4.1+2.7  BMI 75-95%tile:4.1+3.1  BMI 25-75%tile: 4.2+ 3.0  BMI 5-25%tile: 4.3+3.1  BMI <5%tile: 4.7+3.1 | |
| Chiu et al., 2013 | US | | Homeless shelter | CS | 157  70-44.6% boys  87-55.4%girls | 2-17 years | Very high | BMI - CDC 2000  Obesity was defined as a BMI > 95%  on the CDC Children’s BMI Tool for Schools. |  | | As BMI increased, so did caries however the association was not significant (r=0.14, p = 0.08). | |
| **Author, year** | **Country, City** | | **Setting** | **Study design** | **Sample size and gender distribution (n, %)** | **Age group (years)** | **HDI category** | **Definition of weight status** | **Measure of dental caries** | | **Results as reported by the authors** | |
| Chukwumah et al., 2012 | Nigeria, Ugbowo, Benin City | | Private and public schools | CS | 210  Boys:110 (52.4)  Girls:100 (47.6) | 7-15 years | Low | BMI- the participants were categorized into underweight, normal, overweight, and obesity using the BMI percentile set thresholds for children of the same sex and age in CDC growth charts. | FT and DMFT | | DT contributed 90.9% (30/33)  of DMFT that for the mean DMFT in underweight children was 0.21 that for the normal weight children were 0.26 that for the overweight children was 0.50 and for the obese children was  0.18. The prevalence of dental caries was the highest among obese participants followed by normal weight, underweight and overweight participants. There was no significant association between BMI, DMFT and caries experience. | |
| Costa et al., 2013 | Midwest Brazil,  East and North  Health Districts of Goiania in the state of Goias | | Households | CS | 269  Gender distribution not provided | 68.7 ± 3.8 months | High | BMI-for-age z scores  WHO Reference 2007 (ages 5 to19)  .  Children were categorized as severely thin  (<−3SD), thin (<−2SD), normal weight (−2SD to +1SD), overweight (>+1SD, equivalent to BMI  25 kg/m2 at 19 years old), or obese (>+2SD, equivalent to BMI 30 kg/m2 at 19 years old) | dmft Significant Caries Index (SiC) | | BMI was not associated with any of the three categories of dental caries: OR: 1.32 (0.70-2.50);P=0.40. | |
| Costacurta et al., 2011 | Rome, Italy | | Pediatric Dentistry Unit | CS | 107  57 girls (53.3%)  50 boys (46.7%) | 6-12 years | Very high | BMI- Italian population specific charts  Underweight: BMI <3rd centile  Normal weight: 3rd centile < BMI<95th centile  Pre-obese: 75th centile<BMI<95th centile  Obese: BMI>95th centile | DMFT, dmft | | According to the BMI classification, there was no significant association between increase of dmft-DMFT and pre/obesity.  **dmft±SD**  BMI<75^th^ centile: 1.78±1.03  BMI >75^th^ centile:2.23±1.35  **DMFT± SD**  BMI<75^th^ centile:2.31±1.89  BMI >75^th^ centile:2.97±2.18 | |
| De Morais et al., 2010 | Sao Paulo, Brazil | | Public schools | CS | 97  44 boys  53 girls | 8 to12 years | High | BMI percentile- American Academy of Pediatrics  Obese: BMI ≥ 95''' percentile for age;  Overweight: BMI≥ 85''' percentile  and < 95''' percentile;  Normal weight: BMI ≥5^th^ percentile and < the 85''' percentile;  Underweight: BMI <5^th^ percentile. | DMFT, Dmf | | The dental conditions were similar among groups.  **DMFT± SD**  Normal: 0.88±1.33  Underweight: 0.75±1.23  Overweight: 0.97±1.71  **dmft± SD**  Normal: 01.28±1.96  Underweight: 0.67±1.40  Overweight: 0.77±1.48 | |
| **Author, year** | **Country, City** | | **Setting** | **Study design** | **Sample size and gender distribution (n, %)** | **Age group (years)** | **HDI category** | **Definition of weight status** | **Measure of dental caries** | | **Results as reported by the authors** | |
| D’Mello et al., 2011 | Dunedin, New Zealand | | Paediatric Dentistry Clinic | CS | 196  106 (53.0) girls  94 (47.0) boys | 8 years | Very high | BMI -Cole et al 2000  Children were categorised as normal, overweight or obese. | Dmft | | No significant association  was found between the BMI and caries experience (P-value = 0.932). Using the continuous (uncategorized) variables,  there was no significant correlation  between BMI and dmft score (Pearson’s r = )0.06; P = 0.41) or between weight alone and dmft (Pearson’s r = )0.01; P = 0.87) after  adjusting for sex and ethnicity.  Twenty-four percent of the obese children had a dmft score of 8 or more and 37.5% of the overweight children had a dmft of 8 or more. Of the children in the normal weight range, 35.4% had a dmft of 8 or more. | |
| Dye et al., 2004 | USA | | Mobile offices &households | CS | 4236  Boys:2081 (50.8)  Girls:2155 (49.2) | 2-5 years | Very high | BMI-for -age percentiles utilizing age and gender specific CDC BMI percentiles  Overweight: ≥95^th^ percentile  At risk of being overweight: ≥85^th^ < 95^th^ percentile | dfs and ds | | The prevalence of caries did not differ between the three BMI groups  **% caries prevalence (SE)**  BMI ≥95%: 23(3.56)  BMI≥85% <95%: 26.4 (3.00)  BMI <85%: 23.5(1.40) | |
| Edalat et al., 2014 | Iran, Shiraz | | Kindergardens | CS | 202  Boys: 101 (50)  Girls: 101 (50) | 3-6 years | High | BMI-for-age z scores  WHO Reference 2007 (ages 5 to19) and WHO Child Growth Standards (birth to age 5)  The calculations for the two age groups were performed separately  Each record with two standard deviations below the normal value (< -2), was esteemed as abnormal. | dmft | | From children with severe early childhood caries,12.5%were under weight, 5% had height deficiency and 19.5% had BMI deficiency,  however, there was no significant relationship between increasing dmft and BMI deficiency. | |
| Elangovan et al., 2012 | India, Chennai city | | Private, government‑aided, and government schools. | CS | 510  Boys: 266 (52.2)  Girls: 244 (47.8) | 6-12 years | Medium | BMI-for -age percentiles utilizing age and gender specific CDC BMI percentiles  1) underweight (<5thpercentile), 2) normal weight (fifth to 84th percentile),  3) at risk for overweight (>85th to 95th percentile) and  4) overweight (>95th percentile) | deft and DMFT | | Caries prevalence was more in obese children than in other BMI groups. Caries scores increased as BMI‑for‑age increased, though this was not statistically significant.  **mean deft and DMFT**  Underweight (n=113) 1.98±2.735 0.85±0.966  Normal (n=249) 1.78±2.205 0.90±1.137  Overweight (n=88) 1.94±2.246 1.10±1.077  Obese (n=60) 2.55±3.191 1.25±1.385 | |
| **Author, year** | **Country, City** | | **Setting** | **Study design** | **Sample size and gender distribution (n, %)** | **Age group (years)** | **HDI category** | **Definition of weight status** | **Measure of dental caries** | | **Results as reported by the authors** | |
| Granville-Garcia et al., 2008 | Brazil, city of Recife | | Public and private elementary schools | CS | 2651  Unclear if sample included children of both genders | 1-5 years | High | BMI z scores according to WHO criteria  (1989) & National Centre for Health Statistics guidelines.  Children with a Z score > 2 for the weight-height relationship were considered to be  ooverweight. | DMFT | | There was no statistical difference in DMFT score between obese and non-obese children.  **Mean DMFT (SD)**  *Private schools*  Obese:0.298(0.978)  Not obese: 0.493(1.509)  *Public Schools*  Obese:1.161(2.444)  Not obese:1.070(2.236) | |
| Gupta et al., 2014 | Mathura, India | | High schools | CS | 100  50 boys  50 girls | 12 years | Medium | BMI-CDC 2000  Underweight: BMI < 5th percentile.  Healthy weight: BMI 5th percentile to < 85^th^ percentile.  At risk of overweight:BMI 85th to <95th  percentile.  Overweight: BMI ≥95th percentile. | DMFT/dmft | | Statistical analysis by means of a logistic regression model revealed that body mass index had no significant effect on caries prevalence. OR 95%: 0.72(0.365, 1.511; p=0.0411 | |
| Guven Polat et al., 2012 | Turkey | | University pediatric dentistry clinic | CC | 96  43 healthy (19 boys-24 girls)  53 obese (18 boys-35 girls) | Mean for obese: 10.4 years  Mean for healthy: 9.88 years | High | BMI-CDC  Overweight: BMI 85-95^th^ percentile  Obese: BMI>95^th^ percentile | DMFT, dmft | | In both groups, the participants displayed similar DMFT and dmft scores. | |
| Heinrich-Weltzien et al., 2013 | Philippines | | Schools (public elementary) | CS | 1962  Boys: 945 (48.2)  Girls:1017 (51.8) | 6-7 years | Medium | BMI-for-age z scores  WHO Reference 2007 (ages 5 to19)  Overweight: BMI-for-age > 1 SD Obese: BMI-for-age > 2 SDs Underweight: BMI-for-age < 2 SDs | dmft  DMFT  PUFA/pufa index  (P/p), (U/u), (F/f) (A/a). | | Children with a high dmft ratio were more likely to be underweight than children with a lower ratio. However, the relationship was not significant.  **Mean dmft (SD)**  Non underweight: 8.4 (4.2)  Underweight: 8.7 (4.1) | |
| Hong et al., 2008 | USA | | Mobile offices & households | CS | 1507  Boys:48%  Girls: 52% | 2-6 years | Very high | BMI-for -age percentiles utilizing age and gender specific CDC BMI percentiles  Underweight – less than 5^th^ percentile;  Normal weight – 5th percentile  to less than 85th percentile;  At risk of overweight – 85th to less than 95th percentile; Overweight –  equal to or greater than the 95^th^ percentile. | dft | | Generally, children with at-risk BMI or overweight BMI had a higher percentage of caries and higher mean dft than children with normal BMI. Most of these differences across BMI categories were not statistically significant, except for percentages in the 60- <72-month age group *(P =*0.049). BMI ≥95 percentile was not a statistically significant predictor of caries experience OR: 1.20 (0.67-1.03; P=0.08. | |
| **Author, year** | | **Country, City** | **Setting** | **Study design** | **Sample size and gender distribution (n, %)** | **Age group (years)** | **HDI category** | **Definition of weight status** | | **Measure of dental caries** | | **Results as reported by the authors** |
| Jamelli et al., 2010 | | Brazil, Municipality of Caruaru, State of Pernambuco | Public schools | CS and CC study nested within the cross-sectional study. | CS: 689  CC: 647 (465 cases to 182 controls).  Boys: 271 (41.9)  Girls: 376 (58.1) | 12 years | High | BMI percentile –min accordance with the criteria adopted by the Dietary and Nutritional  Surveillance System (SISVAN).  This consisted of a percentile classification of BMI according to age and gender  on the NCHS standard reference scale  Underweight= < P5,  Adequate weight = P5 to < P85, Risk of overweight = v P85 to < P95 and  Overweight/obesity = v P95 | | DMFT | | No statistically significant association was found between these nutritional indices and the occurrence of dental caries. |
| Jurgensen & Petersen, 2009 | | Laos, Vientiane | Secondary Schools | CS | 621  Boys: 293 (47.2)  Girls: 328 (52.8) | 12 years | Medium | BMI- IOTF cut off values and an  empirical method which divided the children into tertiles. | | dmft/DMFT | | No associations were found between Body Mass Index (BMI) and oral health.  Mean DMFT was 1.8 (SEM = 0.09) and caries prevalence was 56%.  dmft-DMFT = 2.3  among normal-weight children with normal weight while dmft-DMFT = 1.6 among overweight children |
| Kopycka-Kedzierawski et al., 2008 | | USA | Mobile offices and households | CS | A total of 10 180 participants  **2-5yrs**:  Boys: 2048/Girls: 2119  **6-11yrs:**  Boys:1502/Girls: 1367  **12-18yrs:**  Boys: 1304/Girls: 1473 | 2-18 years | Very high | BMI-for -age percentiles utilizing age and gender specific CDC BMI percentiles  Overweight: at or above the 95th percentile  At risk for overweight: at or  above the 85th percentile and less than 95th  percentile  Normal weight: less than 85th percentile | | Dfs (2-11 years) and DMFS (6-18 years) | | For children 2–5 years of age, there was no difference in caries experience among normal  weight.  **% with any primary caries (SE)**  Overweight: 23.0(3.6)  At risk: 26.4(3.0)  Normal: 23.5(1.4) |
| Lempert et al., 2014 | | Denmark, Odense | Schools | CS (prospective) | **Baseline**: 385 children  Boys: 178 (46.2)  Girls: 207 (53.8)  **Follow up:** 280 children  Boys: 127 (45.4)  Girls: 153 (54.6) | 8-10 & 14-  16 years | Very high | BMI and BMI z scores- taking age-, sex- and growth- specific considerations into account  National reference z-scores | | dmfs/DMFS | | The linear regression  analysis showed that childhood caries was generally not associated with either BMI or subsequent changes in BMI. Eighty nine of the normal weight children/adolescents were caries free at baseline and 99 at the follow-up examination. The  corresponding numbers for the overweight/obese group were that 12 were caries free at baseline and 13 at the follow-up examination. |
| **Author, year** | | **Country, City** | **Setting** | **Study design** | **Sample size and gender distribution (n, %)** | **Age group (years)** | **HDI category** | **Definition of weight status** | | **Measure of dental caries** | | **Results as reported by the authors** |
| Macek & Milota 2006 | | USA | Mobile offices &households | CS | 7617  (2-5 ys old;  n=1,719)  Permanent dentition  (6-17 ys old;  n=5,898)  Gender distribution not provided | 2 -17 years | Very high | BMI-for -age percentiles utilizing age and gender specific CDC BMI percentiles  Underweight: BMI-for-age less than the fifth percentile;  Normal: BMI-for-age greater than or equal to the fifth percentile and less than the 85^th^ percentile;  At risk of overweight: BMI-for-age greater than or equal to the 85th percentile and less than the 95th percentile; and Overweight: BMI-for-age greater than the 95th percentile. | | dft and DMFT | | Controlling for covariates, there was no significant association between BMI-for-age and dental caries prevalence in either dentition.  **Primary dentition(dft>0,%±SE)**  Underweight:18.0±5.2  Overweight:36.1±6.4  At risk of overweight:26.9±5.0  Normal:28.1±1.8  **Permanent dentition (DMFT>0, %±SE)**  Underweight:31.7±6.3  Overweight:38.8±1.7  At risk of overweight:38.1±2.3  Normal:37.8±1.4 |
| Mapengo et al., 2010 | | Mozambique, Maputo | Government funded schools | CS | 601  Boys: 241 (40.1)  Girls:360 (59.9) | 12 years | Low | BMI-  15th percentile was used  as an indicator of malnutrition and the values above  the 85th percentile were considered overweight indicators and obesity (95^th^) | | DMFT | | The percentage of caries-free adolescents was higher  among children with BMI overweight/obesity (n=48;  69.57%). The prevalence was higher among young  malnourished (n=73; 41.71) when compared to overweight/  obesity (n=21; 30.43%). The differences were not statistically significant (p> 0.05) |
| Mojarad & Maybodi, 2011 | | Western Iran, Hamedan | Private and state elementary schools | CS | 1000  Boys: 500 (50)  Girls: 500 (50) | 6-11 years | High | BMI-for -age percentiles utilizing age and gender specific CDC BMI percentiles  Underweight: under the 5th  percentile curve,  Normal: between 5th and  85th percentile,  At risk for overweight : higher  than 85th and lower than the 95th percentile  Overweight: higher or equal to the 95^th^ percentile | | DFT and dft | | There was not a statistically significant relationship found between high weight and caries frequency in the first (p=0.08)  and permanent dentitions (p=0.06).  Underweight children (n=91) showed a mean DFT value of 1.39 (mean df-t=5.34),  children with normal weight (n=719) had a mean DF-T value of 1.74 (mean df–t=5.1). At  risk of overweight children (n=79) had a mean  DF-T value of 1.59 (mean df- t=4.25), and  overweight children (n=111) had a mean DF-T value of 1.59 (mean df–t=4.79 |
| **Author, year** | | **Country, City** | **Setting** | **Study design** | **Sample size and gender distribution (n, %)** | **Age group (years)** | **HDI category** | **Definition of weight status** | | **Measure of dental caries** | | **Results as reported by the authors** |
| Peng et al., 2014b | | China, Hong Kong | Secondary schools | CS | 514  Boys: 259 (50.4)  Girls: 255 (49.6) | 12 years | High | BMI-for-age z scores  WHO Reference 2007 (ages 5 to19) and BMI Hong Kong Growth Survey weight-for-height criteria | | DMFT | | There was no significant correlation between DMFT  and BMI (r = 0.002, p = 0.96). |
| Peng et al., 2014a | | China, Hong Kong Island | Kindergartens | CS | 324  Boys: 152 (46.9)  Girls: 172 (53.1) | 5 years | High | BMI-for-age z scores and BMI | | dmft | | Dental caries experience was not BMI z score was not associated with dental caries experience. Unadjusted OR 1.30, 95%CI: 0.99, 1.71; P=0.60) Adjusted OR 1.26, 95%CI: 0.94, 1.70; P=0.122) |
| Pinto et al., 2007 | | Pennsylvania, US | Dental school | CS | 135  67 girls  68 males | mean 8.7 years | Very high | BMI –no categories provided, only reference to two previous surveys. | | DS/ds | | No correlation between dental decay in obese and non-obese children was detected (p=0.99).  Mean BMI: 18.36 kg/m2 (SD ±3.5)  Mean Ds score: 2.06% (95% CI 1.4-2.7) |
| Sadeghi & Alizadeh, 2007 | | Iran, Isfahan | Private and state elementary schools | CS | 633  Boys: 317 (50.1)  Girls: 316 (49.9) | 6-11 years | High | BMI-for -age percentiles utilizing age and gender specific CDC BMI percentiles  Underweight: <5thpercentile, Normal weight: fifth to 84th percentile  At risk for overweight: >85th to 95th percentile and  Overweight: >95^th^ percentile | | DFT & dft | | In the normal weight, at risk of overweight, and overweight  groups, the mean ± SE for DFT were 0.34 ± 0.08, 1.23 ± 0.13 and 0.73 ± 0.05, respectively; and 2.01 ± 0.19, 2.76 ± 0.18 and 2.59 ± 0.13 respectively, for dft. Multiple linear regression showed that there was no statistically significant association between BMI-for-age and DFT (R = 0.06) and dft (R = 0.07) indices.  27.7%, 14% and 37.2% of children with normal weight, at risk of overweight and with overweight were caries free, respectively. |
| **Author, year** | | **Country, City** | **Setting** | **Study design** | **Sample size and gender distribution (n, %)** | **Age group (years)** | **HDI category** | **Definition of weight status** | | **Measure of dental caries** | | **Results as reported by the authors** |
| Sadeghi et al., 2011 | | Iran, Rafsanjan, | State and private secondary schools | CS | 747  Boys: 353 (47.3)  Girls: 394 (52.7) | 12-15 years | High | BMI –for- age percentiles  utilizing age and gender specific CDC BMI percentiles  Underweight – less than 5^th^ percentile;  Normal weight – 5th percentile  to less than 85th percentile;  At risk of overweight – 85th to less than 95th percentile; Overweight –  equal to or greater than the 95^th^ percentile. | | DMFT | | There was no significant difference between DMFT scores amongst the BMI-for-age groups (underweight = 2.91, normal-weight = 2.92, at risk of overweight = 2.54, overweight = 2.34, p > 0.05). |
| Scheutz et al., 2007 | | Dar es Salam, Tanzania | Primary schools | Prospective cohort | 218  147  122  Gender distribution not provided | Mean age baseline 7.6 years  Mean age completion: 13.3 | Low | BMI z-scores (Cole et al., 1998)  Malnourished: BMI z-score≤ -1.96 | | DMFS | | Malnutrition at baseline was insignificantly predictive for the development of caries. At baseline mean DMFS was 0.33,  0.37 and 0.32 for cohorts 1, 2, and 3, respectively.  **Adjusted RR, 95% at tooth level:**  Malnourished no (reference)  Malnourished yes: 1.55, 0.92–2.62, p= 0.10 |
| Sheller et al., 2009 | | USA | Children's hospital | Retrospective-CS | 293  Boys: 162 (55)  Girls: 131 (45) | 2-5 years | Very high | BMI –for- age percentiles  utilizing age and gender specific CDC BMI percentiles  Underweight:<5^th^ percentile  Normal:≥5^th^ and <85^th^  At risk of overweight:≥85^th^ and<95^th^  Overweight: ≥95^th^ | | dmft &  the number of pulp-involved  teeth (pulpotomies + pulpectomies + extractions). | | BMI percentile did not correlate with dmft or the number  of pulp-involved teeth.  **Mean dmft (95%CI)**  Underweight: 11.6(10.1-13.1)  Normal: 11.9(11.4-12.4)  At risk of overweight: 11.1 (9.7-12.5)  Overweight: 12.2(10.8-13.6)  **Mean no. of pulp involved**  **Teeth (95% CI)**  Underweight: 4.5 (3.0-5.9)  Normal: 4.0 (3.6-4.5)  At risk of overweight: 4.0 (2.5-5.4)  Overweight: 3.9 (2.9-5.0) |
| **Author, year** | | **Country, City** | **Setting** | **Study design** | **Sample size and gender distribution (n, %)** | **Age group (years)** | **HDI category** | **Definition of weight status** | | **Measure of dental caries** | | **Results as reported by the authors** |
| Sood et al., 2014 | | India | Not reported-Elementary schools? | CS | 280  Boys: 164 (58.6)  Girls: 116 (41.4) | 3-6 years | Medium | BMI –for- age percentiles  utilizing age and gender specific CDC BMI percentiles  Overweight: BMI-for-age at or above 95 percentile  At risk of overweight: BMI for- age between 85th and 95th  Underweight: BMI-for-age less than 5th percentile, and  BMI-for-age between 5th and 85th percentiles under normal weight category.  The BMI-for-age was plotted for each participant on the growth charts as standardized by CDC. Based on these growth charts, the sample population was distributed into three groups:  Group I: Normal weight (5th-85th percentiles)  Group II: Risk of overweight/obese (>85th percentile)  Group III: Underweight (<5th percentile) | | dft | | The differences between the mean dft values among  the three weight status groups were not significant (P = 0.99).  The mean number  of dft in Group I (normal group) was 1.45 ± 3.28. The  mean number of dft in Group II (obese group) was  1.47 ± 2.77. The mean number of dft in Group III  (underweight group) was 1.42 ± 2.68. |
| Tang et al., 2013 | | Kaoshiung, Taiwan | Department of Pediatric Dentistry | CS | 101  38 girls  63 boys | 2-5 years | High | BMI-CDC, Department of Health, Taiwan  Children were classified as obese, overweight, normal or underweight | | SECC | | No statistical significant differences in defs scores among different BMI categories were found (p>0.05).  17.8% of the children with defs scores > 35 were underweight, whereas 11.9% of the children with defs scores <35 were underweight. |
| Tong et al., 2014 | | Leeds, UK | Obese: Pediatric obesity clinic  Community weight managemt group  Normal weight:Orthopaedic Outpatient Fracture Clinic | CC | 64  32 obese  32 normal weight | 7-15 | Very high | BMI-UK 1990 Growth growth charts  Obesity: BMI> 98^th^ percentile | | WHO scoring caries index, DMFT | | There were no statistically significant differences in the DMFT between the BMI groups. |

| **Author, year** | **Country, City** | **Setting** | **Study design** | **Sample size and gender distribution (n, %)** | **Age group (years)** | **HDI category** | **Definition of weight status** | **Measure of dental caries** | **Results as reported by the authors** |
| --- | --- | --- | --- | --- | --- | --- | --- | --- | --- |
| Tramini et al., 2009 | France, Montpellier | Schools | CS | 835  Gender distribution not provided | 12 years | Very high | BMI-IOTF cut off values.  For 12-year-olds these are as follows:  BMI-1 = Insufficient (underweight), under 14.2 kg/m 2 for girls and 14.3 for boys;  BMI-2 = normal weight, between 14.2 and 22 kg/m 2 for girls, between 14.3 and 21.4 kg/m 2 for boys;  BMI-3 = overweight, between 22.1 and 26.6 kg/m 2 for girls, between 21.5 and 26 kg/m 2 for boys;  BMI-4 = obesity, over 26.6 kg/m 2 for girls and over 26 kg/m 2 for boys. | D _3 + 4_ MFT index  DMFT | The mean BMI was  18.9 for the whole sample and the corresponding DMFT value  was 1.47. The best fitted  models for testing the association between BMI and DMFT  were ZIP and ZINB models. They showed a significant asso ciation between DMFT and sugar consumption, but not with  BMI. |
| Tripathi et al., 2010 | India, The city of Bareilly, Uttar Pradesh state | A private and two government schools | CS | 2688  Gender distribution not provided | 6-17 years | Medium | BMI-for-age and gender based on guidelines of National Centre for Health Statistics (1976)  Underweight: < 90%of ideal for  weight/height,  Normal-weight: 90% to110% of  ideal for weight/height,  Overweight: 110% to  120% of ideal weight for height, Obesity: >120% of ideal for weight/height. | DMFT | No correlation between dental decay in obese and non-obese children was detected (p=0.99).  **Mean DMFT (SD)**  Obese: 0.298 (0.980)  Non obese: 0.490 (1.511) |
| Van Gemert-Schriks et al., 2011 | Interior of Surinam | Schools | CS | CS: 380 children  CSBoys: 192 (48.6)  Girls: 188 (51.4) | 6 years | High | BMI standard deviation cores (SDS)  The SDS values were calculated according to Dutch references since there was no growth chart of rainforest children available. | Total-dmfs. Total-ds  Pulpal inflammation was reported  if a carious lesion had reached the pulp and/or when pulpal  exposure was expected on excavation. | No significant correlations between BMI and dmfs were found cross-sectionally.  At baseline, the mean total-dmfs  was 14.0 (±10.1). |
| Vania et al., 2011 | Italy, Rome | Paediatric Dentistry Department of Rome “Sapienza” University | CC | Total: 828  585 ECC (in abstract they say 586) &  243 Caries free  ECC (n= 585):  Boys: 318 (54.4)  Girls: 267 (45.6)  Caries free (n=243): Boys: 117 (48.1)  Girls: 126 (51.9) | 3-6 years | Very high | BMI-for -age percentiles utilizing age and gender specific CDC BMI percentiles  Underweight: <5^th^ percentile  Normal weight: 5^th^ to 84^th^ percentile  At risk for overweight: >85^th^ to <95^th^ percentile  Overweight: > 95^th^ percentile | dmft and the number of teeth with pulp affected by caries. | The distribution of BMI percentiles of the ECC group was: underweight = 10%; normal weight = 55.90%; at risk of overweight = 22.22 %; overweight = 11.11%. The mean BMI percentile of the ECC group was not significantly different from that of the control group. |

| **Author, year** | **Country, City** | **Setting** | **Study design** | **Sample size and gender distribution (n, %)** | **Age group (years)** | **HDI category** | **Definition of weight status** | **Measure of dental caries** | **Results as reported by the authors** |
| --- | --- | --- | --- | --- | --- | --- | --- | --- | --- |
| Werner et al., 2012 | USA, North Carolina | Undergraduate pédiatrie dentistry elinie at the School of Dentistry | CS& retrospective review of a cohort | 230  Boys: 116 (50.4)  Girls: 114 (49.6) | 6- 9 years | Very high | BMI-for -age percentiles utilizing age and gender specific CDC BMI percentiles (for 2-19 years). | DT, dt | The presence of caries in permanent teeth at the initial exam was not significantly different between BMI groups (P=0.41).  **Permanent teeth N(%)**  Underweight/normal:17(11)  Overweight: 6(18)  Obese: 4(14) |
| Wu et al., 2013 | China, Tianjin | Stomatological Hospital of Tianjin Medical University | CS | 280  Boys:151 (53.9)  Girls: 129 (46.1) | 7-12 years | High | BMI- according to BMI classification criteria for obesity of school children in China established by the Chinese Obesity Task Force [2004]-which take into consideration age and gender.  The children were divided into two groups (overweight & normal weight) | DMF-T/S and dmf-t/s | The caries prevalence was 86.3% in the normal-weight group  which was higher than the overweight group (81.0%), but  the difference was not statistically significant (p = 0.24).  The mean DMF-T value for the overweight group and the  normal-weight group was 2.81± 2.61 and 2.98 ± 3.15,  respectively. The difference of the mean DMF-T value between  the overweight group and the normal-weight group  was not statistically significant (p DMF-T = 0.95), similarly  to the mean dmf-t value (p dmf-t = 0.51). The logistic regression model showed no correlation between dental caries and obesity. |
| Xavier et al., 2013 | Southern Brazil,  In the city of Bauru, Midwest region of the state of Sao Paulo | Kindergartens (public) | CS | 229  Gender distribution not provided | 3-5 years | High | BMI-for-age z scores  WHO Reference 2007 (ages 5 to19) | dmft | 66.81% of children presented with nutritional status within the normal range and children with  malnutrition had a mean dmft of 4.0 (3.66), which is two times higher than the other categories of nutritional status.  No statistically significant correlation has been found by correlating dmft and components with Body Mass Index |
| **Author, year** | **Country, City** | **Setting** | **Study design** | **Sample size and gender distribution (n, %)** | **Age group (years)** | **HDI category** | **Definition of weight status** | **Measure of dental caries** | **Results as reported by the authors** |
| Yevenes et al., 2012 | Chile, Metropolitan Region of Santiago | Not reported-assume school | Descriptive transversal, epidemiological  study | 571  Boys: 254 (44.5)  Girls: 317 (55.5) | 6 years | Very high | BMI-for age and gender comparing it to the  CDC-NCHS reference, using the cut-points suggested by  the Health Ministry Technical Norm. | DMFT | No statistically significant difference was found between caries history and nutritional  status (NS) (P=0.837), in both primary and permanent dentition (P=0.306).There was no risk  association (OR 0.88; CI 0.60-1.28; 95%). |
| Yen & Hu, 2013 | Taiwan, Taichung city | Day care centers | CS | 329  Boys: 175 (53)  Girls: 154 (47) | 3-6 years | High | BMI- Based on BMI standards from the Department of Health in Taiwan:  ‘Lean-Underweight  Normal weight  Overweight and  Obese. | deft | After taking into account important factors in the multiple regression models, body mass  index was not significantly associated with dental caries.  **Deft mean ± SD**  Lean: 3.54± 4.54  Underweight: 4.51 ±3.73  Normal:4.68± 3.97  Overweight: 4.73± 4.23  Obese: 3.43 ±3.50 |

*Inconclusive*

| **Author, year** | **Country, City** | **Setting** | **Study design** | **Sample size and gender distribution (n, %)** | **Age group (years)** | **HDI category** | **Definition of weight status** | **Measure of dental caries** | **Results as reported by the authors** |
| --- | --- | --- | --- | --- | --- | --- | --- | --- | --- |
| Mohammadi et al., 2012 | Iran, Kerman | Primary schools | CS | 407  Boys: 223 (54.8)  Girls: 184 (45.2) | 6 years | High | BMI - categorized based  on WHO standards for children at the same age as follows:  BMI < 13.25: underweight for age  BMI 13.26-17.5: normal for age  BMI 17.5-18.55: over weight for age  BMI > 18.5: obese for age | dmft and DMFT | The authors report that there was a significant association between BMI and dental caries categories (p=0.04), however the direction of the association cannot be clarified through the data provided. |

**References**

**Positive relationship**

1. Alm A, Fåhraeus C, Wendt LK, Koch G, Andersson-Gäre B, Birkhed D. Body adiposity status in teenagers and snacking habits in early childhood in relation to approximal caries at 15 years of age. Int J Paediatr Dent. 2008;18(3):189-96.
2. Alm A, Isaksson H, Fahraeus C, Koch G, Andersson-Gare B, Nilsson M, et al. BMI status in Swedish children and young adults in relation to caries prevalence. Swed Dent J. 2011;35(1): 1-8.
3. Bagherian A, Sadeghi M. Association between dental caries and age-specific body mass index in preschool children of an Iranian population. [Indian J Dent Res.](https://www.ncbi.nlm.nih.gov/pubmed/?term=Association+between+dental+caries+and+age-specific+body+mass+index+in+preschool+children+of+an+Iranian+population) 2013; 24(1):66-70.
4. Bener A, Al Darwish MS, Tewfik I, Hoffmann GF. The impact of dietary and lifestyle factors on the risk of dental caries among young children in Qatar.[J Egypt Public Health Assoc.](https://www.ncbi.nlm.nih.gov/pubmed/?term=The+impact+of+dietary+and+lifestyle+factors+on+the+risk+of+dental+caries+among+young+children+in+Qatar) 2013;88(2):67-73.
5. Bhoomika W, Ramakrishna Y, Munshi AK. Relationship between severe early childhood caries and body mass index. J Clin Pediatr Dent. 2013;37(3):235-42.
6. Cantekin K, Gurbuz T, Demirbuga S, Demirci T, Duruk G. Dental caries and body mass index in a sample of 12-year-old eastern Turkish children. J Dent Sci. 2012; 7(1):77-80.
7. dos Santos Junior VE, de Sousa RM, Oliveira MC, de Caldas Junior AF, Rosenblatt A.Early childhood caries and its relationship with perinatal, socioeconomic and nutritional risks: a cross-sectional study. BMC Oral Health. 2014;14:47.
8. Fadel HT, Pliaki A, Gronowitz E, Mårild S, Ramberg P, Dahlèn G, Yucel-Lindberg T, Heijl L, Birkhed D. Clinical and biological indicators of dental caries and periodontal disease in adolescents with or without obesity. Clin Oral Investig. 2014;18(2):359-68.
9. Gerdin EW, Angbratt M, Aronsson K, Eriksson E, Johansson I. Dental caries and body mass index by socio-economic status in Swedish children. Community Dent Oral Epidemiol. 2008;36(5):459-465.
10. Hilgers KK, Kinane DE, Scheetz JP. Association between childhood obesity and smooth-surface caries in posterior teeth: a preliminary study. Pediatr Dent. 2006;28(1):23-8
11. Honne T, Pentapati K, Kumar N, Acharya S. Relationship between obesity/overweight status, sugar consumption and dental caries among adolescents in South India. Int J Dent Hyg.2012;10(4):240-4.
12. Loyola-Rodriguez JP, Villa-Chavez C, Patiñio-Marin N, Aradillas-Garcia C, Gonzalez C, de la Cruz-Mendoza E. Association between caries, obesity and insulin resistance in Mexican adolescents. J Clin Pediatr Dent. 2011;36(1):49-53.
13. Modeer T, Blomberg CC, Wondimu B, Julihn A, Marcus C. Association between obesity, flow rate of whole saliva, and dental caries in adolescents. Obesity (Silver Spring). 2010;18(12):2367-73.
14. Powell JC, Koroluk LD, Phillips CL, Roberts MW. Relationship between adjusted body mass index percentile and decayed, missing, and filled primary teeth. J Dent Child (Chic). 2013;80(3):115-120.
15. Qadri G, Alkilzy M, Feng YS, Splieth C. Overweight and dental caries: the association among German children. Int J Paediatr Dent. 2015;25(3):174-82.
16. Reifsnider E, Mobley C, Beckman Mendez D. Childhood Obesity and Early Childhood Caries in a WIC Population. J Multicult Nurs Health. 2004;10(2): 24-31.
17. Sakeenabi B, Swamy HS, Mohammed RN. Association between obesity, dental caries and socioeconomic status in 6- and 13-year-old school children. Oral Health Prev Dent.2012;10(3):231-41.
18. Shahraki T, Shahraki M, Omrani Mehr S. Association between body mass index and caries frequency among zahedan elementary school children. Int J High Risk Behav Addict. 2013;2(3):122-5.
19. Sharma A, Hegde AM. Relationship between body mass index, caries experience and dietary preferences in children. J Clin Pediatr Dent. 2009;34(1):49-52.
20. Subramaniam P, Singh D. Association of age specific body mass index, dental caries and socioeconomic status of children and adolescents. J Clin Pediatr Dent. 2011;36(2):175-9.
21. Thippeswamy HM, Kumar N, Acharya S, Pentapati KC. [Relationship between body mass index and dental caries among adolescent children in SouthIndia.](https://www.ncbi.nlm.nih.gov/pubmed/22519238) West Indian Med J. 2011;60(5):581-6.
22. Trikaliotis A, Boka V, Kotsanos N, Karagiannis V, Hassapidou M. Short communication: Dmfs and BMI in preschool Greek children. An epidemiological study. Eur Arch Paediatr Dent. 2011;12(3):176-178.
23. Vazquez-Nava F, Vazquez-Rodriguez EM, Saldivar-Gonzalez AH., Lin-Ochoa D, Martinez-Perales GM, Joffre-Velazquez VM. Association between obesity and dental caries in a group of preschool children in Mexico. J Public Health Dent. 2010;70(2):124-130.
24. Willerhausen B, Blettner M, Kasaj A, Hohenfellner K. Association between body mass index and dental health in 1,290 children of elementary schools in a German city. Clin Oral Investig. 2007;11(3):195-200.
25. Willershausen B, Haas G, Krummenauer F, Hohenfellner K. Relationship between high weight and caries frequency in German elementary school children. Eur J Med Res. 2004;9(8): 400-404.
26. Willershausen B, Moschos D, Azrak B, Blettner M. Correlation between oral health and body mass index (BMI) in 2071 primary school pupils. Eur J Med Res. 2007;12(7):295-299.

**Negative relationship**

1. Alkarimi HA, Watt RG, Pikhart H, Sheiham A, Tsakos G. Dental caries and growth in school-age children. Pediatrics. 2014; 133(3): e616-e623.
2. Benzian H, Monse B, Heinrich-Weltzien R, Hobdell M, Mulder J, van Palenstein Helderman W. Untreated severe dental decay: a neglected determinant of low Body Mass Index in 12-year-old Filipino children. BMC Public Health. 2011;11: 558.
3. Cameron FL, Weaver LT, Wright CM, Welbury RR. Dietary and social characteristics of children with severe tooth decay. Scott Med J. 2006 Aug;51(3):26-9.
4. Creske M, Modeste N, Hopp J, Rajaram S, Cort D. How do diet and body mass index impact dental caries in Hispanic elementary school children? J Dent Hyg. 2013;87(1):38-46.
5. Frazão P, Benicio MH, Narvai PC, Cardoso MA.Food insecurity and dental caries in schoolchildren: a cross-sectional survey in the western Brazilian Amazon. Eur J Oral Sci. 2014;122(3):210-5.
6. Freitas AR, Aznar FD, Tinós AM, Yamashita JM, Sales-Peres A, Sales-Peres SH. Association Between Dental Caries Activity, Quality of Life and Obesity in Brazilian Adolescents. Int Dent J. 2014; 64(6): 318-323.
7. Goodson JM, Tavares M, Wang X, Niederman R, Cugini M, Hasturk Het al.Obesity and dental decay: inference on the role of dietary sugar. PLoS ONE. 2013; 8(10):e74461.
8. JahaniY, Eshraghian MR, Foroushani AR, Nourijelyani K, Mohammad K, Shahravan A, Alam M. Effect of socio-demographic status on dental caries in pupils by using a multilevel hurdle model. Health. 2013;5(7):1110-1116.
9. Kopycka-Kedzierawski DT, Auinger P, Billings RJ, Weitzman M. Caries status and overweight in 2- to 18-year-old US children: findings from national surveys. Community Dent Oral Epidemiol. 2008;36(2):157-167.
10. Koksal E, Tekcicek M, Yalcin SS, Tugrul B, Yalcin S, Pekcan G. Association between anthropometric measurements and dental caries in Turkish school children. Cent Eur J Public Health. 2011;19(3):147-151.
11. Narksawat K, Tonmukayakul U, Boonthum A. Association between nutritional status and dental caries in permanent dentition among primary schoolchildren aged 12-14 years, Thailand. Southeast Asian J Trop Med Public Health. 2009;40(2):338-44.
12. Ngoenwiwatkul Y, Leela-adisorn N. Effects of dental caries on nutritional status among first-grade primary school children. Asia Pac J Public Health. 2009;21(2):177-83.
13. Norberg C, Hallström Stalin U, Matsson L, Thorngren-Jerneck K, Klingberg G. Body mass index (BMI) and dental caries in 5-year-old children from southern Sweden. Community Dent Oral Epidemiol. 2012;40(4):315-22.
14. Oliveira LB, Sheiham A, Bönecker M. Exploring the association of dental caries with social factors and nutritional status in Brazilian preschool children. Eur J Oral Sci. 2008;116(1):37-43.
15. Sanchez-Perez L, Irigoyen ME, Zepeda M. Dental caries, tooth eruption timing and obesity: a longitudinal study in a group of Mexican schoolchildren. Acta Odontol Scand. 2010;68(1):57-64.
16. Shakya A, Shenoy R, Rao A. Correlation Between Malnutrition and Dental Caries in Children. J. Nepal Paediatr. Soc.. 2013;33(2):99-102.
17. Sharma A, Hegde AM. Relationship between body mass index, caries experience and dietary preferences in children. J Clin Pediatr Dent. 2009;34(1):49-52.
18. Subramaniam P, Singh D. Association of age specific body mass index, dental caries and socioeconomic status of children and adolescents. J Clin Pediatr Dent. 2011;36(2):175-9.
19. Werner SL, Phillips C, Koroluk LD. Association between childhood obesity and dental caries. Pediatr Dent. 2012;34(1):23-7.

**No relationship**

1. Alm A, Isaksson H, Fahraeus C, Koch G, Andersson-Gare B, Nilsson M, et al. BMI status in Swedish children and young adults in relation to caries prevalence. Swed Dent J. 2011;35(1): 1-8.
2. Alves LS, Susin C, Dame-Teixeira N, Maltz M. Overweight and obesity are not associated with dental caries among 12-year-old South Brazilian schoolchildren. Community Dent Oral Epidemiol. 2013;41(3): 224-231.
3. Chen W. Lack of association between obesity and dental caries in three-year-old children. [Zhonghua Min Guo Xiao Er Ke Yi Xue Hui Za Zhi.](https://www.ncbi.nlm.nih.gov/pubmed/?term=Lack+of+association+between+obesity+and+dental+caries+in+three-year-old+children)  1998; 39(2): 109-111.
4. Chiu SH, Dimarco MA, Prokop JL. Childhood obesity and dental caries in homeless children. J Pediatr Health Care. 2013 Jul-Aug;27(4):278-83.
5. Chukwumah NM, Azodo CC, Adeghe HA, Enabulele JE. Relating dental caries experience with body mass index among Nigerian primary school children: A cross-sectional survey. J Educ Ethics Dent. 2012;2(1):28-32.
6. Costa LR, Daher A, Queiroz, MG. Early Childhood Caries and Body Mass Index in Young Children from Low Income Families. Int J Environ Res Public Health. 2013;10(3): 867-878.
7. Costacurta M, Di Renzo L, Bianchi A, Fabiocchi F, De Lorenzo A, Docimo R. Obesity and dental caries in paediatric patients. A cross-sectional study. Eur J Paediatr Dent. 2011;12(2):112-116.
8. De Morais Tureli MC, de Souza BT, Gavião MB. Associations of masticatory performance with body and dental variables in children. Pediatr Dent. 2010; 32(4):283–288.
9. D'Mello G, Chia L, Hamilton SD, Thomson WM, Drummon BK. Childhood obesity and dental caries among paediatric dental clinic attenders. Int J Paediatr Dent. 2011;21:217–222.
10. Dye BA, Shenkin JD, Ogden CL, Marshall TA, Levy SM, Kanellis MJ. The relationship between healthful eating practices and dental caries in children aged 2-5 years in the United States, 1988-1994. J Am Dent Assoc.2004;135(1):55-66.
11. Edalat A, Abbaszadeh M, Eesvandi M, Heidari A. The Relationship of Severe Early Childhood Caries and Body Mass Index in a Group of 3- to 6-year-old Children in Shiraz. J Dent. 2014;15(2):68-73.
12. Elangovan A1, Mungara J, Joseph E. Exploring the relation between body mass index, diet, and dental caries among 6-12-year-old children. J Indian Soc Pedod Prev Dent. 2012;30(4):293-300.
13. Granville-Garcia AF, de Menezes VA, de Lira PI, Ferreira JM, Leite-Cavalcanti A. Obesity and dental caries among preschool children in Brazil. Rev Salud Publica (Bogota). 2008;10(5):788-795.
14. Gupta P, Gupta N, Singh HP. Prevalence of Dental Caries in relation to Body Mass Index, Daily Sugar Intake, and Oral Hygiene Status in 12-Year-Old School Children in Mathura City: A Pilot Study. Int J Pediatr. 2014; 2014: 1-5.
15. Guven Polat G, Çehreli SB, Tascilar ME, Akgun OM, Altun C, Ozgen IT. The oral health status of healthy and obese children in a Turkish population: a cross-sectional study. Turk J Med Sci. 2012;42(6):970-976.
16. Heinrich-Weltzien R, Monse B, Benzian H, Heinrich J, Kromeyer-Hauschild K. Association of dental caries and weight status in 6- to 7-year-old Filipino children. Clin Oral Investig. 2013;17(6):1515-23.
17. Hong L, Ahmed A, McCunniff M, Overman P, Mathew M. Obesity and dental caries in children aged 2-6 years in the United States: National Health and Nutrition Examination Survey 1999-2002. J Public Health Dent. 2008;68(4):227-33.
18. Jamelli SR, Rodrigues CS, de Lira PI. Nutritional status and prevalence of dental caries among 12-year-old children at public schools: a case-control study. Oral Health Prev Dent. 2014:8(1):77-84.
19. Jürgensen N, Petersen PE. Oral health and the impact of socio-behavioural factors in a cross sectional survey of 12-year old school children in Laos. BMC Oral Health. 2009; 19(29): 1-11.
20. Kopycka-Kedzierawski DT, Auinger P, Billings RJ, Weitzman M. Caries status and overweight in 2- to 18-year-old US children: findings from national surveys. Community Dent Oral Epidemiol. 2008;36(2):157-167.
21. Lempert SM, Froberg K, Christensen LB, Kristensen PL, Heitmann BL. Association between body mass index and caries among children and adolescents. Community Dent Oral Epidemiol. 2014; 42(1):53-60.
22. Macek MD, Mitola DJ.Exploring the association between overweight and dental caries among US children. Pediatr Dent. 2006;28(4):375-380.
23. Mapengo MA, Marsicano JA, Garcia de Moura P, Sales-Peres A, Hobdell M, de Carvalho Sales-Peres SH. Dental caries in adolescents from public schools in Maputo, Mozambique. Int Dent J. 2010;60(4):273-81.
24. Mojarad F, Maybodi MH. Association between dental caries and body mass index among hamedan elementary school children in 2009. J Dent (Tehran). 2011;8(4):170-7.
25. Peng SM, Wong HM, King NM, McGrath C. Association between dental caries and adiposity status (general, central, and peripheral adiposity) in 12-year-old children. Caries Res. 2014;48(1):32-8.
26. Peng SM, Wong HM, King NM, McGrath C. Is dental caries experience associated with adiposity status in preschool children? Int J Paediatr Dent.2014;24(2):122-30.
27. Pinto A, Kim S, Wadenya R, Rosenberg H. Is there an association between weight and dental caries among pediatric patients in an urban dental school? A correlation study. J Dent Educ. 2007;71(11):1435-40.
28. Sadeghi M, Alizadeh F. Association between Dental Caries and Body Mass Index-For-Age among 6-11-Year-Old Children in Isfahan in 2007. J Dent Res. 2007;1(3):119-124.
29. Sadeghi M, Lynch CD, Arsalan A. Is there a correlation between dental caries and body mass index-for-age among adolescents in Iran? Community Dent Health. 2011;28(2):174-7.
30. Scheutz F, Matee MI, Poulsen S, Frydenberg M. Caries risk factors in the permanent dentition of Tanzanian children: a cohort study (1997-2003). Community Dent Oral Epidemiol. 2007;35(6):500-506.
31. Sheller B, Churchill SS, Williams BJ, Davidson B. Body mass index of children with severe early childhood caries. Pediatr Dent. 2009;31(3):216-21.
32. Sood S, Ahuja V, Chowdhry S. Reconnoitring the association of nutritional status with oral health in elementary school-going children of Ghaziabad City, North India.J Indian Soc Pedod Prev Dent. 2014;32(3):197-201.
33. Tang RS, Huang MC, Huang ST. Relationship between dental caries status and anemia in children with severe early childhood caries. Kaohsiung J Med Sci. 2013;29(6):330-6.
34. Tong HJ, Rudolf MC, Muyombwe T, Duggal MS, Balmer R. An investigation into the dental health of children with obesity: an analysis of dental erosion and caries status. Eur Arch Paediatr Dent. 2014;15(3):203-10.
35. Tramini P, Molinari N, Tentscher M, Demattei C, Schulte AG. Association between caries experience and body mass index in 12-year-old French children. Caries res. 2009;43:468-73.
36. Tripathi S, Kiran K, Kamala BK. Relationship between obesity and dental caries in children - A preliminary study. J Int Oral Health. 2010;2(4):65-72.
37. van Gemert-Schriks MC1, van Amerongen EW, Aartman IH, Wennink JM, Ten Cate JM, de Soet JJ. The influence of dental caries on body growth in prepubertal children. Clin Oral Investig. 2011;15(2):141-9.
38. Vania A, Parisella V, Capasso F, Di Tanna GL, Vestri A, Ferrari M, Polimeni A.Early childhood caries underweight or overweight, that is the question. Eur J Paediatr Dent. 2011 Dec;12(4):231-5.
39. Werner SL, Phillips C, Koroluk LD. Association between childhood obesity and dental caries. Pediatr Dent. 2012;34(1):23-7.
40. Wu L, Chang R, Mu Y, Deng X, Wu F, Zhang S, Zhou D. Association between obesity and dental caries in Chinese children. Caries Res. 2013;47(2):171-6.
41. Xavier A, Bastos RDS, Arakawa AM, Caldana MDL, Bastos, JRDM. Correlation between dental caries and nutritional status: preschool children in a Brazilian municipality. Rev Odontol UNESP. 2013;42:378-383.
42. Yevenes I, Zillmann G, Muñoz A, Araya VM, Coronado L, Manríquez J et al. Caries and obesity in 6 year-old schoolchildren from the Metropolitan region (MR) of Santiago, Chile. Rev Odonto Cienc 2012;27(2):121-126.
43. Yen CE, Hu SW. Association between dental caries and obesity in preschool children. Eur J Paediatr Dent. 2013 Sep;14(3):185-9.

**Inconclusive**

1. Mohammadi TM, Hossienian Z, Bakhteyar M. The association of body mass index with dental caries in an Iranian sample of children. Journal of Oral

Health and Oral Epidemiology. 2012;1(1):29-35.
