# Supplementary material for: Body mass index and dental caries in young people: a systematic review
Source: BMC Pediatr. 2019 Apr 23;19:122. doi: 10.1186/s12887-019-1511-x (PMC6480798; doi:10.1186/s12887-019-1511-x)
Supplement: Supplementary file 3 — Risk of flaws in each individual study and across studies. The table contains the risk of flaws between and within studies against all major and minor domains that were evaluated. The meanings of abbreviations are as follow: L = Low risk of flaw; H = High risk flaw; U = Unclear risk flaw; NA = not applicable. (DOCX 71 kb) [file 12887_2019_1511_MOESM3_ESM.docx]

Risk of flaws in each individual study and across studies (L=Low risk, H= High risk, U= Unclear risk, NA= not applicable) *(continued)*

*Studies with a sample size >200*

| Risk of bias domains | Alkarimi et al., 2014 | Alm et al., 2008 | Alm et al., 2011 | Alves et al., 2013 | Bagherian & Sadeghi 2013 | Bener et al., 2013 |
| --- | --- | --- | --- | --- | --- | --- |
| **Exposure definition** | L | L | L | L | L | L |
| **Assessment of exposure** | L | H | H | L | L | L |
| **Assessment of outcome** | L | L | L | L | L | L |
| **Reliability of exposure estimates** | U | U | U | U | L | U |
| **Reliability of outcome estimates** | L | U | U | U | L | U |
| **Confounder assessment** | H | H | H | H | H | H |
| **Sampling bias** | H | L | L | L | L | H |
| **Research specific bias** | L | L | L | L | L | U |
| **Exclusion bias** | U | L | U | L | L | H |
| **Attrition bias** | NA | NA | NA | NA | NA | NA |
| **Funding** | U | U | U | H | U | U |
| **Conflict of interest** | L | U | U | U | U | L |
| **Blinding** | U | U | U | U | U | U |
| **Selective reporting of results** | L | L | L | L | H | L |

| Risk of bias domains | Benzian et al., 2011 | Bhoomika et al., 2013 | Cantekin et al.,2012 | Chen et al., 1998 | Chukwumah et al., 2012 | Costa et al., 2013 |
| --- | --- | --- | --- | --- | --- | --- |
| **Exposure definition** | L | L | L | L | L | L |
| **Assessment of exposure** | L | L | L | L | L | L |
| **Assessment of outcome** | L | L | L | H | L | L |
| **Reliability of exposure estimates** | U | U | U | U | U | U |
| **Reliability of outcome estimates** | L | U | U | U | L | L |
| **Confounder assessment** | H | H | H | H | H | H |
| **Sampling bias** | L | H | H | H | H | L |
| **Research specific bias** | L | H | H | H | H | L |
| **Exclusion bias** | L | L | U | U | U | U |
| **Attrition bias** | NA | NA | NA | NA | NA | NA |
| **Funding** | L | U | U | U | U | U |
| **Conflict of interest** | L | U | U | U | L | L |
| **Blinding** | U | U | L | U | U | U |
| **Selective reporting of results** | H | H | H | H | H | H |

| Risk of bias domains | Dos Santos Junior et al., 2014 | Dye et al., 2004 | Edalat et al., 2014 | Elangovan et al., 2012 | Frazao et al., 2014 | Freitas et al., 2014 |
| --- | --- | --- | --- | --- | --- | --- |
| **Exposure definition** | L | L | L | L | L | L |
| **Assessment of exposure** | L | L | L | L | L | L |
| **Assessment of outcome** | L | L | H | L | L | L |
| **Reliability of exposure estimates** | U | U | U | U | U | U |
| **Reliability of outcome estimates** | L | L | U | U | H | L |
| **Confounder assessment** | H | U | H | H | H | H |
| **Sampling bias** | H | L | H | L | H | H |
| **Research specific bias** | L | L | H | L | L | H |
| **Exclusion bias** | L | U | L | L | L | U |
| **Attrition bias** | NA | NA | NA | NA | NA | NA |
| **Funding** | L | U | U | L | U | U |
| **Conflict of interest** | L | U | L | L | L | L |
| **Blinding** | U | U | U | U | U | U |
| **Selective reporting of results** | L | H | H | L | L | H |

| Risk of bias domains | Gerdin et al., 2008 | Goodson et al., 2013 | Granville-Garcia et al., 2008 | Heinrich-Weltzien et al., 2013 | Hong et al., 2008 | Honne et al., 2012 |
| --- | --- | --- | --- | --- | --- | --- |
| **Exposure definition** | L | L | L | L | L | L |
| **Assessment of exposure** | L | L | L | L | L | L |
| **Assessment of outcome** | L | H | L | L | L | L |
| **Reliability of exposure estimates** | U | U | U | U | U | U |
| **Reliability of outcome estimates** | U | U | L | L | U | L |
| **Confounder assessment** | H | H | H | H | L | U |
| **Sampling bias** | L | H | L | L | L | L |
| **Research specific bias** | L | U | U | L | L | L |
| **Exclusion bias** | U | L | U | U | U | U |
| **Attrition bias** | NA | NA | NA | NA | NA | NA |
| **Funding** | U | L | U | L | U | U |
| **Conflict of interest** | U | L | U | L | U | U |
| **Blinding** | U | U | U | U | U | U |
| **Selective reporting of results** | L | H | L | L | L | H |

| Risk of bias domains | Jahani et al., 2013 | Jamelli et al., 2010 | Jurgensen & Petersen, 2009 | Koskal et al., 2011 | Kopycka-Kedzierawski et al., 2008 | Lempert et al., 2014 |
| --- | --- | --- | --- | --- | --- | --- |
| **Exposure definition** | L | L | L | L | L | L |
| **Assessment of exposure** | L | L | L | L | L | L |
| **Assessment of outcome** | L | H | L | L | L | H |
| **Reliability of exposure estimates** | U | H | U | U | U | U |
| **Reliability of outcome estimates** | L | L | L | U | U | U |
| **Confounder assessment** | H | H | L | H | H | U |
| **Sampling bias** | L | L | L | H | L | H |
| **Research specific bias** | L | L | L | H | L | L |
| **Exclusion bias** | U | U | L | U | L | L |
| **Attrition bias** | NA | NA | NA | NA | NA | NA |
| **Funding** | U | U | U | U | H | U |
| **Conflict of interest** | L | U | L | U | U | U |
| **Blinding** | U | L | U | U | U | U |
| **Selective reporting of results** | L | U | L | H | L | L |

| Risk of bias domains | Macek & Milota 2006 | Mohammadi et al., 2012 | Mapengo et al., 2010 | Mojarad & Maybodi, 2011 | Narksawat et al., 2009 |
| --- | --- | --- | --- | --- | --- |
| **Exposure definition** | L | L | L | L | L |
| **Assessment of exposure** | L | L | L | L | L |
| **Assessment of outcome** | L | L | L | L | L |
| **Reliability of exposure estimates** | U | U | U | U | U |
| **Reliability of outcome estimates** | U | L | L | U | U |
| **Confounder assessment** | H | H | H | H | H |
| **Sampling bias** | L | H | L | H | H |
| **Research specific bias** | L | H | L | H | H |
| **Exclusion bias** | U | L | U | U | U |
| **Attrition bias** | NA | NA | NA | NA | NA |
| **Funding** | U | U | U | U | U |
| **Conflict of interest** | U | L | U | U | U |
| **Blinding** | U | U | U | U | U |
| **Selective reporting of results** | L | H | H | H | L |

| Risk of bias domains | Ngoenwiwatkul & Leela-adisorn, 2009 | Norberg et al., 2012 | Oliveira et al., 2008 | Peng et al., 2014b | Peng et al., 2014a | Powell et al., 2013 |
| --- | --- | --- | --- | --- | --- | --- |
| **Exposure definition** | L | L | L | L | L | L |
| **Assessment of exposure** | L | H | L | L | L | L |
| **Assessment of outcome** | L | U | L | L | L | L |
| **Reliability of exposure estimates** | U | U | U | L | L | U |
| **Reliability of outcome estimates** | U | U | U | L | L | U |
| **Confounder assessment** | H | H | H | U | U | H |
| **Sampling bias** | H | L | H | H | L | H |
| **Research specific bias** | H | L | L | L | L | H |
| **Exclusion bias** | L | L | L | L | L | L |
| **Attrition bias** | NA | NA | NA | NA | NA | U |
| **Funding** | U | U | U | U | U | U |
| **Conflict of interest** | U | U | U | L | L | U |
| **Blinding** | U | U | U | U | U | U |
| **Selective reporting of results** | L | L | L | L | L | L |

| Risk of bias domains | Qadri et al., 2014 | Sadeghi & Alizadeh, 2007 | Sadeghi et al., 2011 | Sakeenabi et al., 2012 | Shahraki et al., 2013 | Shakya et al., 2013 |
| --- | --- | --- | --- | --- | --- | --- |
| **Exposure definition** | L | L | L | L | L | L |
| **Assessment of exposure** | L | L | L | L | L | L |
| **Assessment of outcome** | L | L | L | L | L | L |
| **Reliability of exposure estimates** | U | U | U | U | U | U |
| **Reliability of outcome estimates** | L | U | U | L | H | U |
| **Confounder assessment** | H | H | H | U | H | H |
| **Sampling bias** | H | H | H | L | H | H |
| **Research specific bias** | L | H | H | L | U | H |
| **Exclusion bias** | L | U | U | U | L | U |
| **Attrition bias** | L | NA | NA | NA | NA | NA |
| **Funding** | U | U | U | U | U | L |
| **Conflict of interest** | L | U | L | U | U | L |
| **Blinding** | U | U | U | L | U | U |
| **Selective reporting of results** | L | H | H | L | H | H |

| Risk of bias domains | Sharma & Hedge, 2009 | Sheller et al., 2009 | Sood et al., 2014 | Subramaniam & Singh, 2011 | Thippeswamy et al., 2011 | Tramini et al., 2009 |
| --- | --- | --- | --- | --- | --- | --- |
| **Exposure definition** | L | L | L | L | L | L |
| **Assessment of exposure** | L | H | L | L | L | L |
| **Assessment of outcome** | L | L | L | L | L | L |
| **Reliability of exposure estimates** | U | U | U | U | U | U |
| **Reliability of outcome estimates** | H | L | U | L | L | U |
| **Confounder assessment** | H | H | H | U | U | U |
| **Sampling bias** | H | H | H | H | H | L |
| **Research specific bias** | H | H | H | H | L | L |
| **Exclusion bias** | U | U | U | U | L | U |
| **Attrition bias** | NA | NA | NA | NA | NA | NA |
| **Funding** | U | U | L | U | U | U |
| **Conflict of interest** | U | U | L | U | U | U |
| **Blinding** | U | U | U | U | U | U |
| **Selective reporting of results** | H | L | H | H | L | L |

| Risk of bias domains | Trikaliotis et al., 2011 | Tripathi et al., 2010 | Van Gemert-Schriks et al., 2011 | Vania et al., 2011 | Vazquez-Nava et al., 2010 | Werner et al., 2012 |
| --- | --- | --- | --- | --- | --- | --- |
| **Exposure definition** | L | L | H | L | L | L |
| **Assessment of exposure** | L | L | L | H | L | H |
| **Assessment of outcome** | L | L | L | L | L | H |
| **Reliability of exposure estimates** | U | U | U | U | U | U |
| **Reliability of outcome estimates** | L | L | L | U | L | U |
| **Confounder assessment** | H | H | H | H | H | H |
| **Sampling bias** | H | H | H | H | H | H |
| **Research specific bias** | H | H | H | H | H | H |
| **Exclusion bias** | U | U | U | U | U | L |
| **Attrition bias** | NA | NA | NA | NA | NA | NA |
| **Funding** | H | L | U | U | U | U |
| **Conflict of interest** | U | L | L | U | U | U |
| **Blinding** | U | L | U | U | U | U |
| **Selective reporting of results** | H | H | H | H | L | H |

| Risk of bias domains | Willerhausen et al., 2007 | Willershausen et al., 2004 | Willershausen et al., 2007 | Wu et al., 2013 | Xavier et al., 2013 | Yevenes et al., 2012 | Yen & Hu, 2013 |
| --- | --- | --- | --- | --- | --- | --- | --- |
| **Exposure definition** | L | L | L | L | L | L | L |
| **Assessment of exposure** | L | L | L | L | L | L | L |
| **Assessment of outcome** | L | L | H | L | L | L | L |
| **Reliability of exposure estimates** | U | U | U | U | U | U | U |
| **Reliability of outcome estimates** | U | U | U | H | L | L | U |
| **Confounder assessment** | H | H | H | H | H | H | H |
| **Sampling bias** | H | H | H | H | L | L | H |
| **Research specific bias** | H | H | H | L | L | L | H |
| **Exclusion bias** | L | U | U | U | U | U | H |
| **Attrition bias** | NA | NA | NA | NA | NA | NA | NA |
| **Funding** | U | U | U | U | U | U | U |
| **Conflict of interest** | U | U | U | L | L | L | U |
| **Blinding** | U | U | U | U | U | U | U |
| **Selective reporting of results** | L | H | H | L | H | H | L |

*Studies with a sample size <200*

| Risk of bias domains | Cameron et al., 2006 | Chiu et al., 2013 | Costacurta et al., 2011 | Creske et al., 2013 | De Morais et al., 2010 | D’Mello et al., 2011 |
| --- | --- | --- | --- | --- | --- | --- |
| **Exposure definition** | H | L | L | L | L | L |
| **Assessment of exposure** | L | L | L | L | L | H |
| **Assessment of outcome** | H | L | H | H | L | H |
| **Reliability of exposure estimates** | U | U | U | U | U | U |
| **Reliability of outcome estimates** | U | L | U | U | L | U |
| **Confounder assessment** | U | H | H | U | H | H |
| **Sampling bias** | H | H | H | H | H | H |
| **Research specific bias** | H | H | H | L | L | H |
| **Exclusion bias** | U | U | U | U | U | U |
| **Attrition bias** | NA | NA | NA | NA | NA | NA |
| **Funding** | U | U | U | U | U | U |
| **Conflict of interest** | U | U | U | U | U | U |
| **Blinding** | U | U | U | U | U | U |
| **Selective reporting of results** | U | L | L | L | L | L |

| Risk of bias domains | Fadel et al., 2014 | Gupta et al., 2014 | Guven Polat et al., 2012 | Hilgers et al., 2006 | Loyola et al., 2010 | Modeer et al., 2010 |
| --- | --- | --- | --- | --- | --- | --- |
| **Exposure definition** | L | L | L | L | L | L |
| **Assessment of exposure** | L | L | U | H | L | L |
| **Assessment of outcome** | L | L | H | H | L | H |
| **Reliability of exposure estimates** | U | U | U | L | U | U |
| **Reliability of outcome estimates** | L | U | U | L | L | U |
| **Confounder assessment** | H | H | H | H | H | H |
| **Sampling bias** | H | U | H | H | H | H |
| **Research specific bias** | L | L | H | H | H | H |
| **Exclusion bias** | U | U | U | U | L | U |
| **Attrition bias** | L | NA | U | NA | L | U |
| **Funding** | U | U | U | U | U | H |
| **Conflict of interest** | L | L | U | U | U | U |
| **Blinding** | U | U | U | L | L | U |
| **Selective reporting of results** | L | L | L | L | H | L |

| Risk of bias domains | Pinto et al., 2007 | Reifsnider et al., 2004 | Sancez et al., 2010 | Scheutz et al., 2007 | Tang et al., 2013 | Tong et al., 2014 |
| --- | --- | --- | --- | --- | --- | --- |
| **Exposure definition** | H | H | L | L | L | L |
| **Assessment of exposure** | H | H | L | L | L | L |
| **Assessment of outcome** | H | H | L | L | H | L |
| **Reliability of exposure estimates** | U | U | L | U | U | U |
| **Reliability of outcome estimates** | H | U | L | L | U | L |
| **Confounder assessment** | H | H | H | H | H | H |
| **Sampling bias** | H | H | H | U | H | H |
| **Research specific bias** | H | H | H | H | H | L |
| **Exclusion bias** | U | U | U | U | U | U |
| **Attrition bias** | NA | NA | L | H | NA | H |
| **Funding** | U | U | L | U | L | U |
| **Conflict of interest** | U | U | L | U | U | L |
| **Blinding** | L | U | L | U | U | U |
| **Selective reporting of results** | L | U | L | L | L | L |
